# MDCT Imaging of Non-Traumatic Thoracic Aortic Emergencies and Its Impact on Diagnosis and Management—A Reappraisal

**DOI:** 10.3390/tomography8010017

**Published:** 2022-01-13

**Authors:** Tullio Valente, Giacomo Sica, Giorgio Bocchini, Federica Romano, Francesco Lassandro, Gaetano Rea, Emanuele Muto, Antonio Pinto, Francesca Iacobellis, Paola Crivelli, Ahmad Abu-Omar, Mariano Scaglione

**Affiliations:** 1 Department of Radiology, Monaldi Hospital, Azienda Ospedaliera dei Colli, 80131 Naples, Italy; sicagf@libero.it (G.S.); giorgio.bocchini@gmail.com (G.B.); fedinromano@gmail.com (F.R.); f.lassandro@gmail.com (F.L.); gaetano.rea71@gmail.com (G.R.); emanuelemuto@gmail.com (E.M.); 2 Department of Radiology, CTO Hospital, Azienda Ospedaliera dei Colli, 80131 Naples, Italy; antopin1968@libero.it; 3 Department of Radiology, Cardarelli Hospital, 80131 Naples, Italy; iacobellisf@gmail.com; 4 Department of Radiology, University of Sassari, 07100 Sassari, Italy; paocri2000@gmail.com (P.C.); scaglionefun@gmail.com (M.S.); 5 Department of Radiology, James Cook University Hospital, Middlesbrough TS4 3BW, UK; aomar@doctors.org.uk; 6 Department of Radiology, Pineta Grande Hospital, Pineta Grande, 81030 Castel Volturno, Italy

**Keywords:** aorta, acute aortic syndrome, aortic aneurysm, aortic emergencies, aortic dissection, imaging of the aorta, emergency CT, computed tomography angiography, TEVAR, management

## Abstract

Non-traumatic thoracic aorta emergencies are associated with significant morbidity and mortality. Diseases of the intimomedial layers (aortic dissection and variants) have been grouped under the common term of acute aortic syndrome because they are life-threatening conditions clinically indistinguishable on presentation. Patients with aortic dissection may present with a wide variety of symptoms secondary to the pattern of dissection and end organ malperfusion. Other conditions may be seen in patients with acute symptoms, including ruptured and unstable thoracic aortic aneurysm, iatrogenic or infective pseudoaneurysms, aortic fistula, acute aortic thrombus/occlusive disease, and vasculitis. Imaging plays a pivotal role in the patient’s management and care. In the emergency room, chest X-ray is the initial imaging test offering a screening evaluation for alternative common differential diagnoses and a preliminary assessment of the mediastinal dimensions. State-of-the-art multidetector computed tomography angiography (CTA) provides a widely available, rapid, replicable, noninvasive diagnostic imaging with sensitivity approaching 100%. It is an impressive tool in decision-making process with a deep impact on treatment including endovascular or open surgical or conservative treatment. Radiologists must be familiar with the spectrum of these entities to help triage patients appropriately and efficiently. Understanding the imaging findings and proper measurement techniques allow the radiologist to suggest the most appropriate next management step.

## 1. Introduction

**Figure 1:**
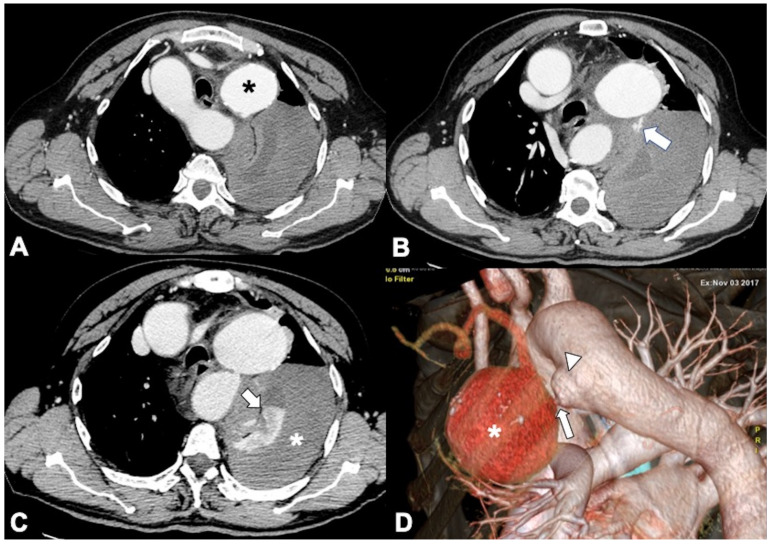
Computed tomography angiography (CTA) provides a rapid noninvasive diagnosis of the entire aorta including aberrant anatomy and pathology. (**A**) A 74-year-old man presenting in ED with 10 h of abrupt onset of acute chest pain, improving anemia, hypotension (blood pressure of 70/40 mm Hg in both upper extremities), and shortness of breath. CTA shows a right aortic arch and a pleural and extrapleural left hematoma from an aneurysm (asterisk) of a retroesophageal aberrant left subclavian artery (ALSA). (**B**) More caudal axial scan shows the ALSA aneurysm rupture site (arrow). Note the anomalous course of the left brachiocefalic vein posterior to the ascending aorta. (**C**) CTA-delayed phase shows contrast medium extravasation in the extrapleural hematoma (asterisk); note extrapleural fat sign (arrow). (**D**) 3D volume rendering oblique sagittal reformat image clearly shows the aneurysm (asterisk) coming immediately off the ALSA origin (arrow) from a Kommerell’s diverticulum (arrowhead).

Acute non-traumatic thoracic aorta emergencies (TAE) often present with similar clinical symptoms, and thereby investigation of the appropriate management algorithm is heavily reliant on radiological findings [1,2]. These non-traumatic pathologies include life-threatening conditions caused by acute diseases of the thoracic aorta (Table 1). In the Emergency Department (ED), plain chest radiography is the initial imaging investigation offering a screening evaluation for alternative common differential diagnoses, a preliminary assessment of the anomalous aortic contours/mediastinal dimensions, and occasionally a porcelain aorta [3,4,5]. In fact, >20% of patients with confirmed acute aortic dissection (AAD) lacked mediastinal or aortic contour abnormalities [6]. Its poor sensitivity and specificity means it has a limited role in definitive diagnosis [4,5,6]. Although 2D echocardiography (TTE) is the first imaging technique to evaluate patients with thoracic pain in the emergency room, but it is often limited by artifacts and permits adequate assessment mainly of some proximal aortic segments. The key role of TTE is its ability to rapidly assess cardiac complications of aortic diseases such as aortic valve insufficiency, pericardial fluid, wall motion abnormalities, and pleural fluid. If incomplete aortic information is present, other imaging techniques must be considered. State-of-the-art multidetector-computed tomography angiography (CTA) provides a widely available, rapid, replicable, noninvasive, multiplanar imaging with excellent diagnostic performance (sensitivity/specificity 98–100%), offering a detailed visualization of the entire aorta, aortic branches including aberrant anatomy, and its surrounding structures (Figure 1) [7,8]. It is the gold standard for aortic imaging and plays a pivotal role in the patient’s management and care [9]. Diagnosis and treatment entail a multidisciplinary (aorta team) approach involving some diagnostic methods, and medical, endovascular, or surgical interventions. The aim of this review was to provide an overview of imaging of acute non-traumatic aortic pathology, particularly emphasizing the role of the state-of-the-art CTA imaging, the clues to diagnosis, and the impact on variable management strategies.

### 1.1. Thoracic Aorta Anatomy: Key Points of Imaging

The aorta is a unique organ, both in its structure and function. In a person’s lifetime, the heart may beat up to 3000 million times and pump some 200 million liters of blood [10]. During diastole, the systolic-stored potential energy of the elastic aortic wall is converted into kinetic energy returned as elastic recoil sustaining flow to coronary arteries and peripheral vessels (Windkessel effect, allowed by the thoracic aortic media that contains approximately 53–78 lamellar units vs. approximately 28 units of the abdominal aortic wall) [11,12]. Therefore, the aorta is much more than a hollow, passive conduit [13]. It is the prototype “elastic artery” (elastic pipe) featuring an incredibly thick tunica media rich in smooth muscle and elastic fibers. In fact, the aorta is so thick that it requires its own nutritive capillary network: the vasa vasorum. Five segments of the thoracic aorta can be differentiated: the intrapericardial root (from annulus to sinuses of Valsalva until sinotubular junction); the ascending aorta; the extrapericardial aortic arch extending from the brachiocephalic trunk (BCT) to the left subclavian artery (LSA); the isthmus (site of attachment of the ligamentum arteriosus); and the descending thoracic aorta (DTA). In the redefined zonal map anatomy, the division of four anatomic zones of the thoracic aorta is widely accepted to help choose between open surgery and complex endovascular approach [14,15]. Imaging key points of the anatomy of aorta are listed in Table 2.

**Table 2:** Thoracic aorta anatomy: Imaging considerations and normal measurements.

The aortic *intima* is in direct contact with blood and has anti-thrombotic and anti-atherosclerotic functions [16]. The *media* is mainly responsible for aortic wall elasticity. The *adventitia* provides blood supply to the aortic wall and surrounding nerves through the vasa vasorum.
Intrapericardial *root* consists of the annulus and three sinuses that allow the well coaptated valve leaflets to open to 90°; sinuses normal diameter = 34.2 ± 4.1 mm (2 standard deviations (SD)) ^1^.
Anomalies and variations of coronary artery ostia are fairly common.
The root motion (1.5 cm/cardiac cycle) confers artifacts to non-gated imaging with low temporal resolution.
The STJ ^2^ is normally a well-defined site (waist-like contour) at which the rounded sinuses join the narrower tubular-shaped ascending aorta; normal diameter = 29.7 ± 3.4 mm (2 SD) ^1^
*Ascending* is the intrapericardial segment of the aorta from the STJ to the first arch great vessel; normal diameter 32.7 ± 3.8 mm (2 SD); accepted maximal diameter < 40 mm ^1^.
The ascending aorta and pulmonary trunk have a common adventitia at the root of the great vessels; extravasated blood from the ascending aorta can extend beneath the adventitia of the pulmonary trunk and cross the barrier of the pulmonary hilum.
The transverse *arch* is the extrapericardial section of the aorta between the ascending aorta and DTA ^3^, extending from the brachiocephalic trunk to the left subclavian artery; these two vessels offer a natural barrier that can stop the extension of the dissection. A classical 3-vessel arch configuration is seen in about 70% of patients.
Anomalies and variation in aortic arch configuration are fairly common (30% of patients).
Arch orientation is from right to left and from anterior to posterior and shows different age- and sex-related angulation; it has a greater or outer curvature (convexity) and an inner or smaller curvature (concavity).
Arch 3D complex curvature makes it a place of significant turbulence, endothelial dysfunction, and early severe atherosclerotic transformation.
*Isthmus* is the short portion of the aorta just distal to the origin of the left subclavian artery at the site of the ligamentum arteriosum; it is the preferential site of trauma injury, coarctation, and Type B dissection.
Ductus diverticulum is an anatomical variant at the site of the aortic isthmus, a developmental outpouching of the thoracic aorta that may be mistaken for an acute aortic pseudoaneurysm and that needs to be known.
*DTA*^3^ is the left paraspinal vertically oriented aortic portion from the isthmus to the level of the diaphragmatic aortic hiatus; normal diameter 17–26 mm (95% CI) (end diastolic); accepted maximal diameter ≤ 30 mm ^1^.

^1^ Larger variations are appropriated for sex, age, phase of heart cycle, body surface area, and BMI. ^2^ STJ = sinotubular junction. ^3^ *DTA* = descending thoracic aorta.

### 1.2. CTA Technique

Advances in scanner technology have established CTA as a first-line imaging modality in the evaluation of aortic disease.

Image-acquisition protocols are finalized to obtain homogeneous contrast material enhancement, while minimizing aortic wall motion, radiation dose, and intravenous contrast material volume. The rapid evolution of fast CT scanners such as 16 to 320 detector rows allowed isotropic three-dimensional volumetric data acquisition, excellent multiplanar reconstruction (MPR), curved planar reconstruction, maximum-intensity projection (MIP), endoluminal views, and volume rendering (VR) of the entire aorta [17,18].

CTA protocols for aortic evaluation can use either non-gated or gated techniques. ECG-gated examinations minimize cardiac motion artifacts and can be performed either retrospectively by using a helical technique or prospectively by using a step and shoot acquisition. Additionally, dual-source scanners can use an ultrafast high-pitch helical mode (flash mode) to generate motion-free images of the aortic root with lower-radiation exposure [19,20]. Due to the ultrafast acquisition, the entire aorta can be scanned in just 3 s. Additional evaluation of the coronary arteries as part of a CTA study require prospective or retrospective ECG-gating.

**Figure 2:**
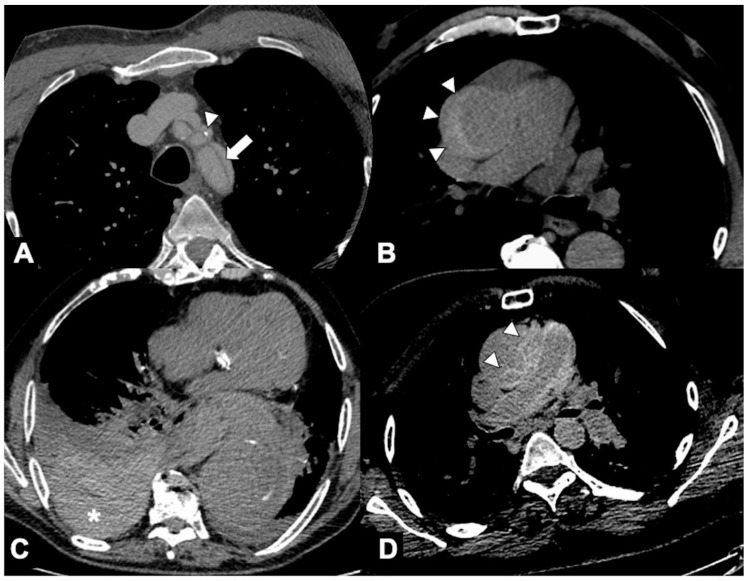
Usefulness of CTA low-dose unenhanced phase in acute aortic syndrome. (**A**) Noncontrast CT axial scan shows dissection flap (arrow) and displaced intimal calcifications (arrowhead) in supra-aortic vessels extension of type A aortic dissection in a 79-year-old-man with acute neck and back pain. (**B**) Axial image from noncontrast CT shows a crescent-shaped high-density rind in the ascending aorta, a typical appearance of type A acute intramural hematoma (arrowheads). (**C**) Noncontrast CT axial scan shows a large bilateral pleural effusion with increased density in the dependent aspect of the collection (hematocrit sign), highly suggestive of hemothorax (asterisk) from rupture of the descending thoracic aorta aneurysm. (**D**) Axial image of aorta from noncontrast CT shows hemomediastinum tracking along the main and right pulmonary arteries and hyperattenuating dissection flap (arrowheads) in ascending aorta in a 68-year-old woman with ruptured type A dissection.

A complete CTA protocol includes three phases: unenhanced, arterial, and delayed phases. The exact protocol for each patient will vary, depending on the vendor and patient body habitus [21,22]. Our protocol of CTA study consisted in:

(1) A low-dose, non-gated, non-contrast phase, with coverage from the lung apex to the lower abdomen, which is useful to evaluate the presence of aortic intramural hematoma, displaced intimal calcification, surgical material, and high-density pericardial, pleural, or mediastinal blood (Figure 2). The radiation dose of the non-contrast phase should be kept low by using wider collimation and low tube potential with concomitant reduction in the tube current. Nowadays, dual-source technology provides a virtual non-contrast phase obviating the need to acquire a non-enhanced phase.

(2) An ECG-gated arterial (or alternatively ultra-high pitch) scan-acquisition using automated 80–110 mL of 370 mg I/mL iodinated contrast material (CM) or 60–80 mL of 400 mg I/mL iodinated contrast according to the patient body weight, by pump injectors delivered at a rate between 3.5 and 5 mL/s to achieve a target opacification of the aorta of 250 HU. It may be performed with bolus tracking or utilization of a timing bolus to ensure optimal enhancement of the great vessels. Particularly by using retrospective-gating, the CT scanner is acquiring during the entire cardiac cycle; therefore, it is possible to correct dysrhythmias or cardiac motion artifacts. Since retrospective ECG-gating is associated with a significant increase in radiation dose, various dose-reduction techniques may be used, such as prospective ECG triggering, ECG-based tube current modulation, automatic exposure control, lower peak kilovoltage, and iterative/deep-learning reconstruction algorithms.
(3) A non-gated delayed phase (about 70 s delay) obtained to assess for late filling of a false lumen (FL), to clearly depict abdominal organ malperfusion, contrast extravasation due to aortic rupture and to evaluate peri-adventitial enhancement indicating acute aortitis.

The CTA imaging technique is appropriate and crucial for an accurate diagnosis.

## 2. Acute Aortic Syndrome (AAS): Dissection and Variants

Interrelated diseases sharing intimal and medial layer disruption (aortic dissection and variants according to Svensson’s classification) have been grouped under the umbrella term of acute aortic syndrome because they are life-threatening conditions clinically indistinguishable on presentation [22,23,24,25,26]. Their incidence varies from 3.5 to 6.0/100,000 patient-years and can occur as isolated phenomena or in tandem. AAS is defined as “acute” within 14 days of the onset of symptoms. All mechanisms that weaken the media layer of the aorta (some hereditary conditions like Marfan’s syndrome and other genetic connective tissue diseases) or increases the aortic wall’s shear stress (poor controlled hypertension in primis) are high-risk features and can eventually result in an AAS [22,23,24].

**Figure 3:**
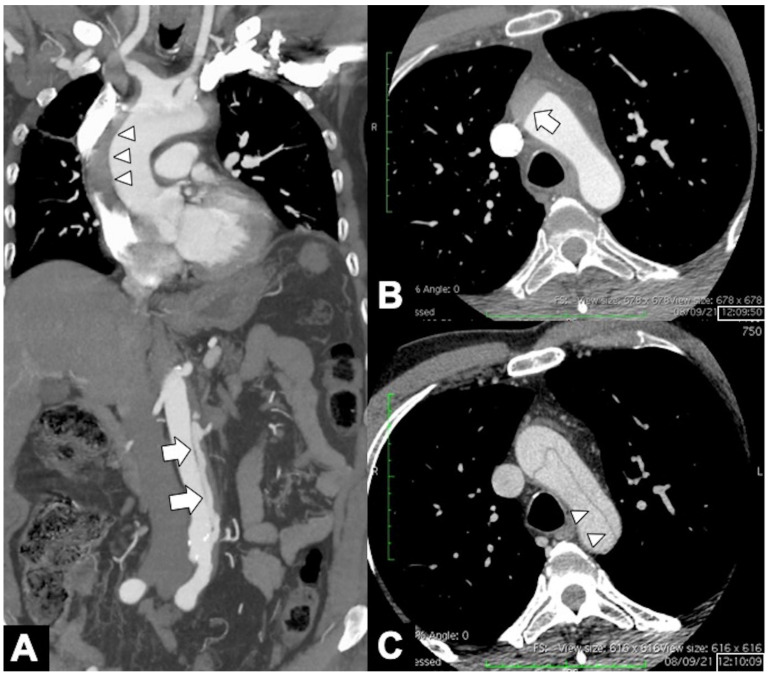
(**A**) Coronal MPR image of CTA in a 57-year-old man with chest pain and troponin negative shows coexistence of an acute intramural hematoma in the ascending aorta (type A IMH, arrowheads) and a dual-barrel dissection in the abdominal aorta (arrows). (**B**,**C**) Axial CTA images in another patient, showing the extension of the dissection flap from the proximal arch (arrow) to its distal segment (arrowheads) after only 19 s in the same CTA examination. Aortic dissection may be a very dynamic process.

**Figure 4:**
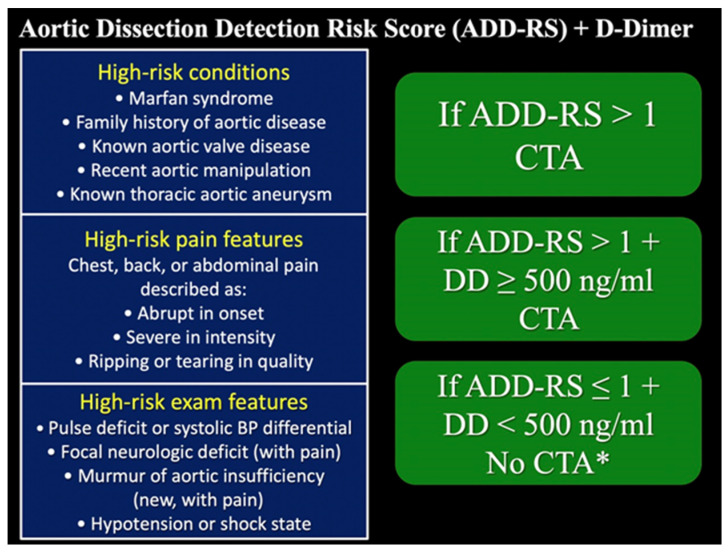
Pretest clinical probability assessment according to Aortic Dissection Detection Risk Score (ADD-RS) and D-Dimer (DD). ADD-RS score is based on 12 risk factors organized in three categories (0, low risk; 1, intermediate risk; 2–3. high risk). BP = blood pressure. * Caution in patients with early presentation (≤2 h) or long-lasting symptoms (≥1 week) [28].

The clinical connection between these entities is defined by the typical, abrupt onset and severe chest or back “aortic” pain often in elderly patients who are poor surgical candidates having multiple comorbidities including coronary artery disease, hypertension, and diabetes [22,23,24,25,26,27]. These associated processes are characterized by dynamic damage to the different components of the aortic wall (Figure 3A) and may coexist, rendering it impossible to know which came first (Figure 3B,C). The aortic dissection validated detection risk score (ADD-RS) is a clinical decision tool that aids in grading the pretest probability of an acute aortic dissection to reduce the rate of misdiagnosis, ranging from 0 to 3 [25,28,29]. In the ADvISED Prospective Multicenter Study, the integration of ADD-RS = 0 or ≤1 with D-dimer (DD) < 500 ng/mL shows negligible statistical heterogeneity and consistently high sensitivity, thus supporting reliability for the diagnostic rule-out of AAS (Figure 4) [28]. However, this score has a low specificity, and the lack of defined inclusion criteria limits the application of this rule in practice. Recent research suggests that the methodology for this score development was sub-optimal, and it has not been robustly validated in the emergency department chest pain population [30]. Further prospective studies are necessary to reduce the risk of selection bias and to confirm the validity of the score. The International Registry of Acute Aortic Dissection (IRAD) was established in 1996 for the purpose of enrolling many patients at several aortic centers to assess the presentation, management, and outcome of Aortic Dissection (AD) [6,31]. A fast diagnosis and referral of the patient to a specialized hospital with an aortic team can be the difference between life and death [32]. It is imperative for radiologists to find a common language with the clinicians whilst reporting morphologic features in AAS [33]. A complete AAS reporting list is shown in Table 3.

### 2.1. Classic Acute Aortic Dissection (AD): Pathology and Typical and Atypical Imaging Findings

**Figure 5:**
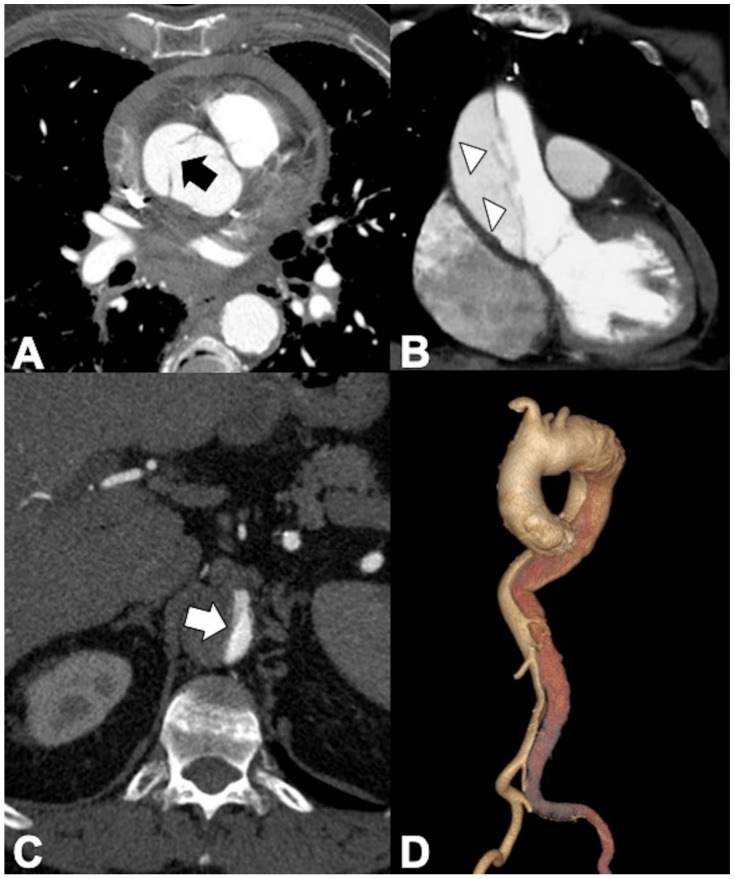
CTA findings in aortic dissection. (**A**) Axial CTA image showing intimomedial rupture sign in the ascending aorta (arrow) in type A dissection, indicating direction of the intimomedial entrance tear from true to false lumen. (**B**) Coronal CTA MPR image showing the intimomedial flap travelling parallel to the aorta long axis. Note the cobweb sign by thin, string-like filaments of the media layer in the false lumen (arrowheads). (**C**) Axial CTA image shows pressure competition between lumina and opacified true lumen collapse (arrow). (**D**) The different density of the TL (more intensely opacified due to faster flow) and FL in the early angiographic phase allows them to be clearly distinguished in the coronal VR reconstruction.

AD (70% of AAS) arises from a tear in the aortic intima (primary or most proximal entry tear) exposing the medial layer to the pulsatile blood flow. CTA intimomedial rupture sign enables direct visualization of the intimomedial rupture indicating direction of the intimomedial entrance tear from true (TL) to false lumen (FL) in systole. However, the direction can be bidirectional or reversed depending on the cardiac phase (Figure 5A) [34].

The most proximal tear usually occurs at a point of high shear force in systole (lateral right wall of ascending aorta, distal to LSA in the DTA). The progressive separation of the aortic wall layers results in the formation of a FL within the media having pressures ≥ those in the TL, which travels parallel to the aorta long axis (Figure 5B) [21,24,25,35]. The inner layer of the media remains contiguous with the aortic intima, creating an intimomedial flap, readily seen on cross-sectional imaging [21,24,25,36,37].

**Figure 6:**
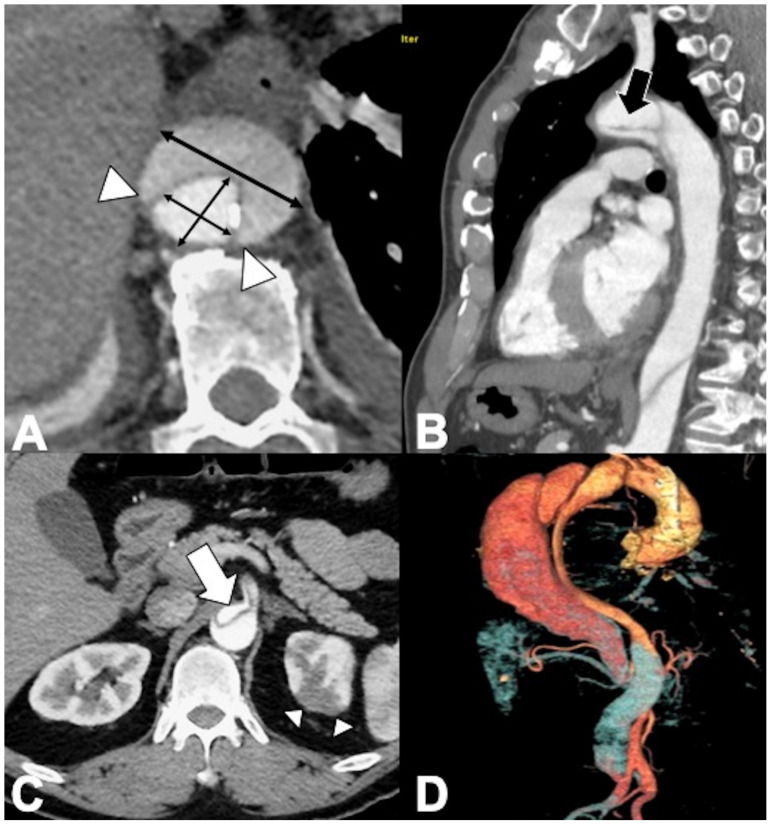
CTA findings in classic dual-barrel aortic dissection. (**A**) Axial CTA images showing the “beak sign” (arrowheads) in the false lumen because its wall and intimomedial flap usually form acute angles resembling a bird’s beak. In the radiological report, maximum aortic diameter and minimum and maximum aortic true lumen diameter should be included. (**B**) Sagittal oblique MPR image of non-A-non-B dissection with descending-entry and retrograde arch extension (arrow) in a 75-year-old male presenting with acute chest pain. (**C**) Axial CTA images. Static aortic dissection is seen as protrusion of the intimal flap (arrow) into the ostium of the affected branch vessel causing subsequent partial or total thrombosis of the branch vessel with resulting perfusion impairments. Note left kidney partial infarct (arrowheads). (**D**) CTA oblique coronal VR reconstruction image shows a three lumina aortic dissection.

The outer portion of the media remains attached to the adventitial layer, creating the thin weak outer wall of the FL that dilates. The greater the proportion of the media involved in the flap, the thinner the external wall of the FL, and therefore, the higher the risk of adventitial disruption and aortic rupture [36,37,38]. The dissections may remain patent as a false lumen, thrombose, recommunicate with the true lumen through fenestrations (re-entry tears), or rupture into potential spaces such as the pericardial, pleural, or peritoneal cavities. Aortic rupture quickly leads to exsanguination and death. The overall degree of dilation of the FL depends on the blood pressure, the residual wall thickness, and the percentage of wall circumference involved in the dissection. One or more re-entry communications between lumina allow blood redirection into the TL and free blood circulation between lumina. Progressive pressurization of the FL resulting in TL collapse is highly variable based on the number of tears between the lumina, the chronicity of the flap, and the degree of control of the patient’s blood pressure (Figure 5C) [36,37,38]. Conversely, the FL may also end blindly in a cul-de-sac, creating a blood clot. Differentiation between TL and FL is important in the planning of percutaneous treatment with endovascular grafts or surgical repair of aortic dissections [39]. The main CTA findings that differentiate TL and FL are listed in Table 4 (Figure 5D) [36,37,38,39]. The beak sign occurs at the acute angle of the intimomedial flap and outer wall of the FL (Figure 6A) [40]; the cobweb sign represents strands or ribbons of media crossing the FL and appearing as thin filiform filling defects [39,41]. Calcification may help to differentiate between the FL and TL—it has been described as only being seen in the wall of TL [25,37]. The FL (and the flap) can propagate distally toward the aortic bifurcation and retrograde toward the aortic root (Figure 6B).

Aortic branches static (in most cases caused by expansion of the FL into a branch vessel) or dynamic (dissection flap covers vessel origin during systole) occlusion can lead to obstruction of arterial branches and malperfusion syndromes (Figure 6C) [17]. A dynamic obstruction can be intermittent or persistent and may be managed by increasing FL outflow with fenestration of the intimal flap, while a static obstruction or ostial disruption should be treated by stenting of the malperfused branch vessel. Different clinical and imaging combinations of dissecting membrane extension patterns and malperfusion syndromes represent multiple facets of this pleomorphic disease, and each combination can lead to a dramatically different outcome [42].

Atypical features of the intimal flap are due to dissection of the entire intima resulting in a circumferential flap (windsock sign) that may be partially or totally separated from the aorta (intimointimal intussusception), with the detached tissue forced downstream [43]. Furthermore, the intussuscepted flap can obstruct the aortic arch and its branches. Other findings are an extremely narrow filiform TL, calcified FL in chronic dissection, and a three-channel aorta (Mercedes–Benz sign) with several False lumens (Figure 6D) [44].

### 2.2. AD: Classifications, Predictors of Mortality, and Management Options

The AD management-based Stanford and DeBakey classifications were introduced over 50 years ago and have an important prognostic value, being decisive to treatment [45,46].

In the emergency radiology setting, Stanford classification is preferred because it dictates immediate clinical management: Surgical for type A involving the ascending aorta versus medical for type B involving the DTA beyond LSA origin, regardless of the site of the entry tear [24,25]. In general, the treatment and diagnosis recommendations for all AAS are like AD based on Stanford classification. Recently, a new subdivision type of Stanford classification emerged, trying to address the aortic arch dissections, called non-A/non-B type, which is an aortic arch dissection not accompanied by the involvement of the ascending aorta, with or without involvement of the descending thoracic or abdominal aorta [47,48,49].

#### 2.2.1. Type A (TAAD)

TAAD is the most common type of AD, accounting for 62% of patients, according to the International Registry of Acute Aortic Dissection (IRAD) [6]. Because it carries a high mortality (mortality rate around 1–2% per hour in the first 48 h; in-hospital mortality rate from 22 to 31%) is a surgical emergency, and surgical repair must be performed immediately after the diagnosis [50]. When not surgically repaired (medical mortality 57%), poor prognosis TAAD can progress to aortic rupture and cardiac tamponade or to coronary/cerebral malperfusion and myocardial infarction (MI), the aforementioned being the most common causes of death. Clinically unstable patients, age ≥ 70 years, previous cardiac surgery, hypotension or shock at presentation, any pulse deficit, and an ECG finding indicative of myocardial ischemia or infarction are all preoperative predictors of mortality [50,51]. Retrograde type A dissection comprises 7–25% of acute type A dissections [52].

**Figure 7:**
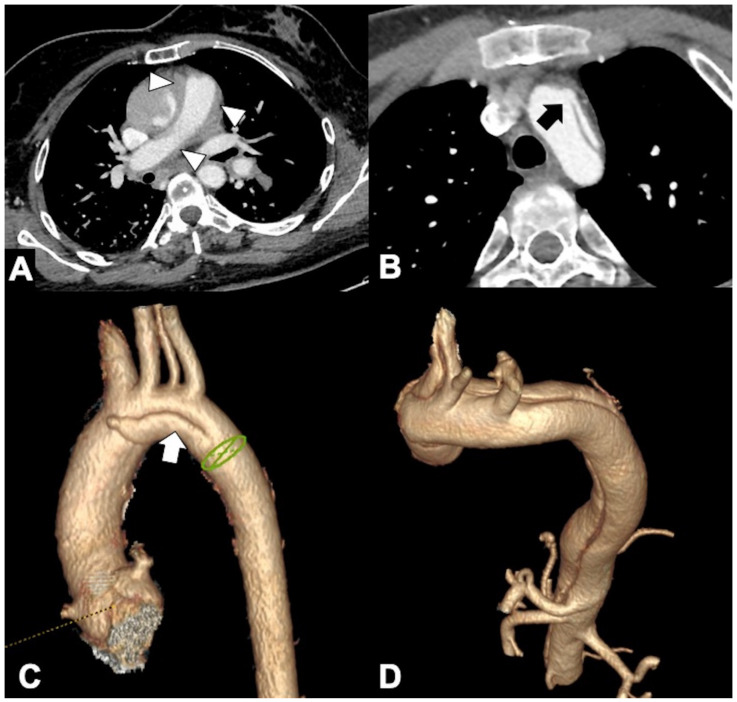
(**A**) Axial CTA image demonstrates thickening of the main pulmonary arterial wall (arrowheads) indicating IMH of the PA in ruptured Type A AD. (**B**) Axial CTA image shows entry tear (arrow) in the arch convexity in a 53-year-old man with severe chest pain. (**C**) 3D-VR parasagittal reconstruction image shows localized 4-vessels arch dissection (arrow) without supra-aortic trunks involvement. (**D**) 3D-VR parasagittal reconstruction image shows a non-A-non-B dissection with arch-entry and anterograde descending aorta involvement.

The root of the ascending aorta shares an adventia with the pulmonary artery [53,54]. When there is a dissection (or a ruptured aneurysm) of the ascending aorta, blood can accumulate and exert pressure on the pulmonary artery causing a hematoma of the pulmonary artery that crosses the barrier of the pulmonary hilum extending into the pulmonary interlobular septa and alveoli depicted as Ground Glass opacities (Figure 7A) [55,56,57,58,59,60].

#### 2.2.2. Type B (TBAD)

According to its initial manifestation, TBAD can be classified as complicated or uncomplicated. Acute complicated TBAD is defined by the presence of at least one of the following signs: Aortic rupture, refractory pain, rapid aortic expansion and an aneurysm > 55 mm, uncontrollable hypertension (persisting despite commencement of three different classes of antihypertensive therapy), visceral, renal or limb malperfusion, paraplegia/paraparesis (spinal malperfusion), and periaortic hematoma, which worsen the prognosis and increase mortality [24,25,61,62]. In these patients, thoracic endovascular aortic repair (TEVAR) is the first-line therapy in favorable anatomy because it has well-known appealing qualities including a minimally invasive procedure with rapid deployment and decreased operative time and blood loss [24,25]. The treatment goal of TEVAR is to seal the proximal intimal tear with a stent graft and depressurize the FL by redirecting flow into the TL. For patients with non-complicated TBAD, optimal medical therapy alone reducing aortic-wall stress caused by high blood pressure, a high heart rate, and ventricular contraction is essential and must be adopted. Sometimes in real life defining TBAD as uncomplicated can be challenging. Up to 25% of the initially uncomplicated TBAD develops late complications, in a median interval of 7 days after onset of symptoms and must undergo repeat CT imaging and TEVAR [24,25,63,64,65]. Predictive factors for late complications of TBAD are the early dilatation of the thoracic aorta > 40 mm, the presence of a FL diameter > 22 mm, a proximal entry tear size ≥ 10 mm, a severe TL compromise, as well as its location at the inner aortic curvature or close to the left subclavian artery. On the other hand, in uncomplicated TBAD, the use of pre-emptive TEVAR rather than medical therapy alone to prevent late complications, while intuitive, requires further study in randomized cohorts [66].

#### 2.2.3. Type Non-A/Non-B AD

Its incidence is still not well known, but in some studies, it accounts for up to 11% of patients with acute AD [67]. Based on CTA findings, it is possible to categorize three different non-A/non-B AD anatomical configurations:

- A type B AD, with primary ET distal to the LSA and extension of the flap both antegrade into the descending thoracic and/or abdominal aorta both retrograde into the arch (B retrograde with arch extension; in some cases, retrograde intramural hematoma extension) [47,66,67,68,69];
- The primary ET is located in the arch, and the dissection flap is limited to the arch (arch-alone) (Figure 7B,C).
- The primary ET is in the arch, and the flap propagates in the distal aorta, without involvement of the ascending aorta (Figure 7D).

For Type non-A/non-B AD, open (frozen elephant trunk/FET surgery) or endovascular intervention paired with debranching may be options. Management of non-A non-B dissections remains challenging, and our knowledge on the subject is still limited [47,49,67,68,69].

### 2.3. Acute Intramural Hematoma (IMH)

IMH is defined as a small size (≥5 mm) hematoma in the media of the aortic wall without visible intimal tear and intimomedial flap [24,70,71,72]. It ranges from 5 to 27% between AAS and can spontaneously occur as an isolated process (90%) or can be found in association with penetrating atherosclerotic ulcer (PAU) (5%) or as a post-traumatic or iatrogenic aortic injury [23,24,31,70]. PAU-associated IMH has a significantly worsened prognosis with a higher risk of expansion and rupture [71]. Krukenberg first proposed that rupture of the vasa vasorum initiated the process of aortic dissection in 1920 [73]. However, there is increasing recognition based on surgery and autopsy findings (and recently also on imaging due to improved technology and resolution) that IMH may result from microscopic intimal tears, often not detectable by imaging studies. Currently, there is a belief that IMH is a variant or precursor of AD [74,75,76,77,78]. IMH is more frequently observed in the DTA (Type B IMH, 60–70%) and less commonly in the ascending aorta and arch (Type A IMH; 30% and 10%, respectively) [79]. Essentially, the aortic wall layers are separated and filled with thrombus (maybe because IMH has an entry tear only without a re-entry site) rather than free-flowing blood of a classic dissection, and those cases labeled as IMH are actually cases of acute AD or AD with an acutely occluded and thrombosed FL (a non-communicating type of aortic dissection) [78].

**Figure 8:**
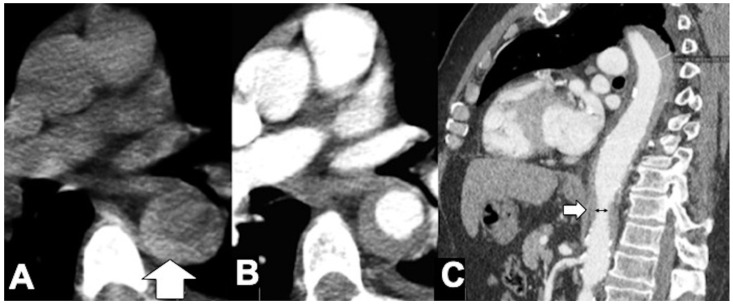
CT features of acute type B IMH in a 62-year-old man with abrupt chest pain and Type B IMH at baseline CTA examination. (**A**) Axial low-dose unenhanced CT image shows hyperdense crescent sign of the aortic wall (arrow). (**B**) Axial arterial phase CTA image in the same patient shows decreased diameter of the aortic lumen and the smooth luminal-wall interface. (**C**) Sagittal reconstruction CTA image shows decreased diameter of the aortic lumen (arrow) and a relative constant circumferential relationship with the wall.

Normal aortic wall thickness is <3 mm. Unenhanced CT images are pivotal in establishing the diagnostic hallmark of IMH depicted as a non-spiral, hyperattenuating (60 ± 15 HU) wall thickening of ≥5 mm in an eccentric or concentric pattern [22,37]. There may be central displacement of intimal calcification and a degree of vascular luminal narrowing, but the luminal–wall interface remains smooth (Figure 8). By definition, IMH has neither an intimal flap nor double channel intraluminal flow. The hematoma remains similar in appearance on CTA, with no enhancement of the thickened wall. The longitudinal extent of the IMH can be very short (~1 cm) or can extend the full length of the aorta. In the case of type A IMH, aortic-wall thickening creates a separation between the aortic lumen and the right atrial appendage that normally does not exist [80]. It is important to report the minimum and maximum transverse diameters of the aortic lumen at the level of the hematoma to stratify the risk of progression. Involvement of the ascending aorta (Type A IMH) carries a high in-hospital mortality (up to 40%). On the other hand, Type B IMH is less likely to be associated with an adverse outcome, with an in-hospital mortality risk of <10%. The natural history of IMH is highly variable and can be difficult to predict. The prognosis and management of IMH is further dependent on several key morphologic prognostic features, which may be recognized with CTA serial imaging and are listed in Table 5 [81,82,83].

**Figure 9:**
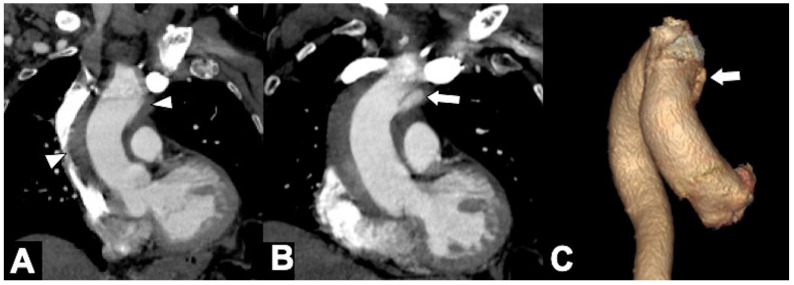
Unstable Type A IMH complicated by ulcer-like projection (ULP). (**A**) Coronal oblique CTA shows type A IMH (arrowheads) in a 68-year-old man with hypertension and chest pain. The total aortic diameter was less than 50 mm and the IMH thickness less than 11 mm; he underwent medical management initially. (**B**) Three-day follow-up coronal oblique CTA image shows disease progression by an ULP due to opening of intimal tear (arrow). (**C**) 3D-volume-rendered (VR) reconstruction confirms ULP (arrow).

Malperfusion syndromes are less common in IMH patients than in AD, but periaortic hematoma and subsequent pericardial effusion are more common. After the initial detection and management of IMH, serial follow-up imaging is accomplished with CT or MRI to document resolution, stability, or progression. IMH conversion to dissection often occurs within 3–8 days (Figure 9). Development of an aneurysm (saccular or fusiform) is indicative of progressive weakening of all three layers of the aortic wall. The most frequent long-term complication of IMH is the development of aortic fusiform aneurysm secondary to structural weakness of the media, usually in the subacute or chronic stages of the disease [80,81,82]. A focal intimal disruption represented by contrast material-filled small pouch (<3 mm) projecting outside the opacified aortic lumen must always be sought on the baseline CTA; it can quickly become an ulcer-like projection (ULP) with a >3 mm communicating neck, which can progress to a frank double-barrel dissection. ULP is distinguished from PAU in that it typically is not present at the initial CT but is identified at follow-up imaging and may occur in patients with no evidence of atherosclerotic disease [84,85,86]. The appearance of ULP in the acute phase has a poor prognosis, particularly when it is in the ascending aorta or aortic arch, and frequently progresses to dissection, saccular pseudoaneurysm, or rupture. Intramural blood pool (IBP) or aortic branch artery pseudoaneurysm is a focal contrast pooling (hematoma) measuring at least 10 mm in thickness and arising along the nonpleural circumference of the aorta; communicating with and typically located at the origin of an aortic side branch (e.g., bronchial, intercostal, intercostobronchial, pericardial and lumbar artery), it has a very small (<2 mm) or imperceptible communication to the aortic lumen [21,24,25]. IBPs do not adversely affect prognosis, often regress spontaneously, and require no dedicated treatment [21,24,25,81].

### 2.4. Penetrating Atherosclerotic Ulcer (PAU)

**Figure 10:**
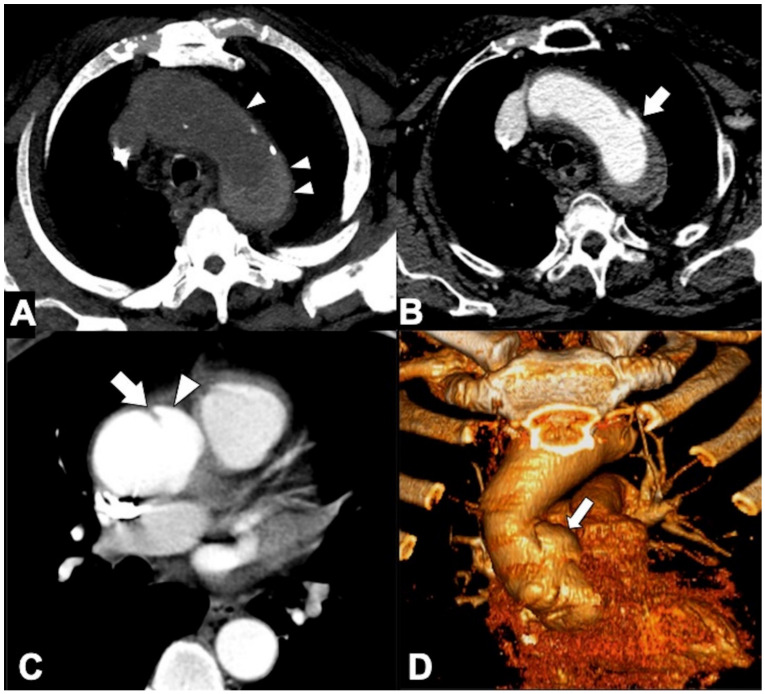
CTA features of Svensson’s Class 4 (PAU) and Class 3 (limited intimal tear) variants. (**A**) Axial unenhanced CT image shows aortic arch high-density intramural hematoma (IMH) in a 74-year-old male with acute neck and chest pain (arrowheads). (**B**) CTA axial scan in the same patient shows a flat penetrating ulcer (arrow) with associated IMH in the arch convexity. (**C**) CTA axial image shows a linear filling defect with subtle undermined edges (arrow) and eccentric medial one-sided bulge of the ascending aorta (arrowhead) without a clear intimomeadial flap or false lumen in a 58-year-old male with sudden onset of chest and back pain. (**D**) 3D-VR oblique coronal reconstruction confirms the eccentric one-sided saccular aortic bulging (‘‘mushroom cap’’ morphology) of class 3 dissection (arrow).

In PAU, an atherosclerotic plaque ulcerates, eroding through the internal elastic lamina and extending into the aortic media, indicating markedly diseased intima seen in the setting of advanced atherosclerosis [87]. It accounts for 2–7% of AAS. It is typically seen in elderly individuals with multiple other comorbidities and occurs more commonly along the DTA. On CTA, PAU manifests as a localized ulcer (as an outpouching with jagged margins) with adjacent subintimal hematoma (IMH) on a background of extensive atheromatous changes. Risk factors at imaging are PAU diameter and depth (width > 2 cm and depth > 1 cm) and the presence of saccular aneurysm [85,86,87]. Isolated PAU, PAU with IMH, and PAU with saccular aneurysm are three different PAU presentations that can be demonstrated on CTA (Figure 10A,B) [88]. Most PAUs are asymptomatic and do not require urgent invasive treatment. However, it may be a progressive disease leading to a saccular (pseudo)aneurysm, AD, or transmural aortic rupture (40% of symptomatic PAUs), and emergency invasive therapy (often TEVAR) is recommended [21,24,25,89,90,91].

### 2.5. Limited AD (Class 3 Dissection Variant)

Limited intimal tears (LITs) of the aorta (Class 3 dissection variant) are the least common (~3%) form of pathology in the dissection spectrum [21,92,93,94,95]. LIT, or incomplete dissection, limited dissection, or intimal tear without a hematoma is a spontaneous laceration of the aortic intima and subjacent media without significant dissection of blood into the media and the double-barrel lumen of classic AD [94]. LITs are difficult to detect on imaging and often are underappreciated or missed [96,97,98]. On high-quality CTA, the lacerated edges of an incomplete dissection retract, and the residual “bare area” may be seen as a linear filling defect (focal flaps) without a clear dissection flap, often associated with a small volume of localized intramural hematoma under the lesion edges, together showing a focal aneurysmal dilatation or outward aortic contour bulging [21,95,96,97]. Extravascular findings include pleural and pericardial effusion, mediastinal hematoma, hemothorax, hemopericardium, periaortic stranding or hematoma, pulmonary artery sub-adventitial hematoma, and esophageal abnormality. Post-processing techniques, particularly 3D volume-rendered slabs and endoluminal views, can help identify and display the extent of these lesions, which can occur anywhere in the thoracic aorta (Figure 10C,D). The underlying pathology, natural history, and risk of rupture imply a treatment approach similar to AD and IMH [24,25].

## 3. Unstable and Impending Rupture of Thoracic Aorta Aneurysm (TAA)

Thoracic aortic aneurysm (TAA) is the focal dilatation of the thoracic aorta to more than 1.5 times its normal diameter. The true aneurysm is covered by all three layers of the arterial wall, is usually associated with fusiform (spindle-shaped) dilatation of the aorta, and mostly occurs as a consequence of atherosclerotic disease (in up to 70% of cases, but 20% of degenerative aneurysms have saccular morphology) [99,100,101].

Several genetic, inflammatory, anatomic (bicuspid aortic valve is an independent risk factor for TAA formation), drug-abuse, and medical-clinical conditions (e.g., hypertension) may be associated with TAA [102]. Aneurysms are largely asymptomatic prior to complications, and treatment of stable disease has better outcomes that treatment of acute presentation.

**Figure 11:**
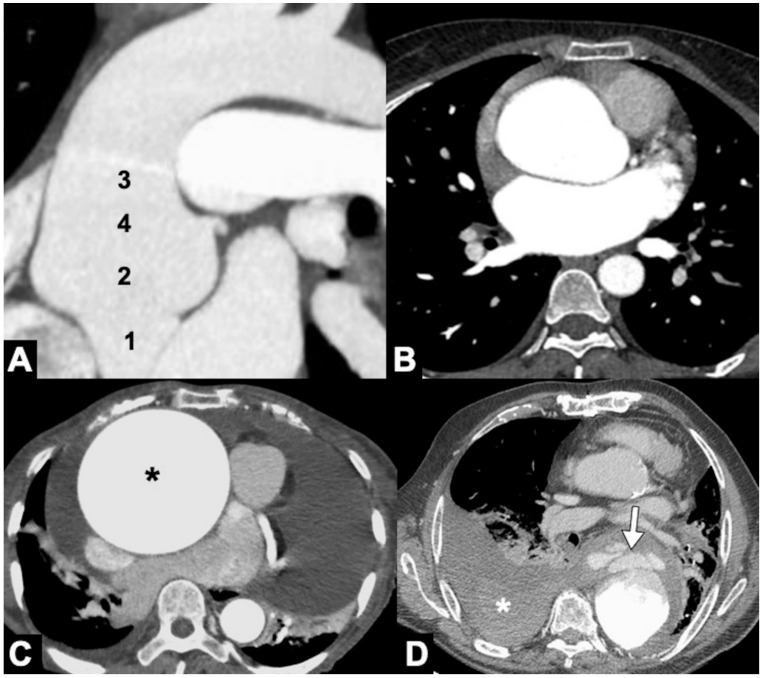
CTA of thoracic aorta aneurysms. (**A**) Marfan syndrome, a multisystem connective tissue disease caused by a defect in the protein fibrillin 1, and annuloaortic ectasia in a 42-year-old man. Sagittal MIP reconstruction image shows a proximal dilatation of the aortic anulus (**1**), sinuses of Valsalva (**2**) along with effacement of the STJ (**4**) producing a pear-shaped ascending aorta (**3**). (**B**) Axial CTA image shows sinuses ectasia in the same patient. In Marfan syndrome, a cut-off value of 5 cm of the ascending aorta diameter is recommended for surgical repair. (**C**) Axial CTA image shows a 12 cm ascending aorta aneurysm (asterisk) and a large mediastinal effusion. The risk of rupture of TAAs increases with size of the aneurysm according to Laplace’s law. (**D**) Axial CTA image shows a ruptured atherosclerotic aneurysm of the descending thoracic aorta. Note the high-attenuation fluid in the right pleural space, representing acute hemothorax (asterisk), and contrast medium extravasation from the aortic lumen (arrow).

**Figure 12:**
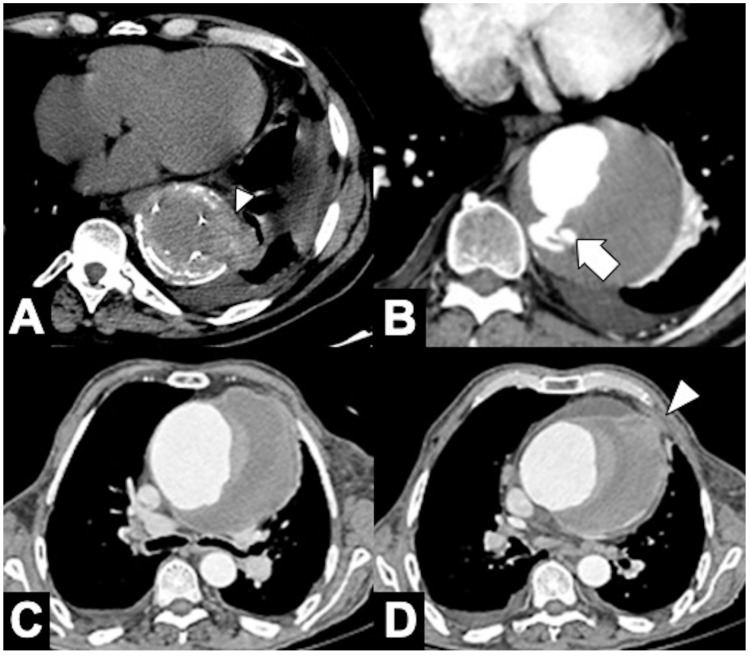
CTA findings in impending rupture of the thoracic aorta aneurysms. (**A**) Missing calcium sign (arrowhead). (**B**) Thrombus fissuration (arrow). (**C**) Hyperattenuating crescent sign, periaortic stranding sign, and contour irregularity of the aneurysm. (**D**) Periaortic fat stranding blurs a focal area of the mediastinum and extends to the chest wall representing an inflammatory process (arrowheads) [111].

Because an abdominal aortic aneurysm occurs in 28% of patients with a TAA, it is important that the initial evaluation includes the entire thoracoabdominal aorta. TAAs are classified by location as affecting more commonly the ascending aorta (36%), aortic arch (34%), or DTA (30%) [103]. Annuloaortic ectasia is a condition with dilated sinuses of Valsalva along with effacement of the sinotubular junction (STJ) producing a pear-shaped ascending aorta commonly seen associated with Marfan syndrome and other connective tissue disorders (Figure 11A,B). The Crawford classification modified by Safi describes five types of thoraco-abdominal aortic aneurysms [104]. In the evaluation of TAA, CTA accurate depiction of aortic caliber on centerline imaging, morphology, relationship to the aortic arch vessels and the presence of thrombus or ulceration are of importance in deciding whether and how to intervene [104,105]. Accurately measured maximal aortic diameter is currently the primary metric used to guide surveillance strategy and the timing of surgical/endovascular intervention for patients with TAA (Figure 11C). The generally accepted aneurysm growth rate is 4 mm/year, and intercurrent dissection is associated with a more rapid growth rate. When the size of the aorta reaches its biomechanical “hinge point,” usually about 5.5 cm in diameter for ascending aorta and 7 cm for DTA, wall integrity rapidly declines, growth accelerates, and the incidence of complications rapidly increases. A rupture occurs when mechanical stresses on the wall exceed the wall strength, and as dictated by the law of Laplace, the rupture risk increases with aneurysmal size (Figure 11D) [106,107]. Clinical subtle and non-specific symptoms may already correspond to clear radiological signs of impending rupture. As far as the most accepted widespread criteria are represented by the maximum diameter and the expansion rate, there are other important imaging features that radiologists must be aware of, to promptly detect potentially life-threatening conditions (Table 6). These features are considered by most authors as signs of impending rupture (SIR) and include, amongst others, (1) a hyper-attenuating crescent sign, (2) focal wall discontinuity of circumferential calcifications (Figure 12A), (3) aortic bulges or blebs, and (4) a draped aorta sign or periaortic stranding [108,109]. These signs are mostly based on CT characteristics of the mural thrombus. Aneurysms are indeed often characterized by a thrombus lining the walls and by a patent lumen. As some authors consider the width of the mural thrombus a protective factor for aneurysm stability, the reduction in its width is considered a criterion of higher rupture risk [110]. Amongst the aforementioned SIR, the “hyperattenuating crescent sign” results from intraluminal blood creating a fissure that transits through the unstable mural thrombus to the intimal margin, forming a typical semilunar shape [111].

Discontinuity of thrombus circumferential calcification, also known as a “missing calcium sign,” refers to the focal interruption of mural calcification, indicating the site of impending rupture, especially if the aortic lumen tapers toward the focal discontinuity (tangential calcium sign). It is worth mentioning that these are the commonest signs associated with asymptomatic patients who developed sudden symptoms later [21]. Aortic bulging or blebs and contour irregularity of the aneurysm refer to the focal bulging of the aneurysm wall, which generally indicates loss of elastic fibers and inflammatory changes that underlie a potential rupture [112]. This sign together with the “draped aorta sign,” in which there is loss of the normal fat plane between the aneurysm and the vertebral bodies, with a characteristic wavy shape of the posterior aortic wall, are considered less-dangerous findings with no rupture demonstrated on follow-up imaging in most cases. On the other hand, periaortic fat stranding can indicate extraluminal rupture (Figure 12C,D) [112,113,114].

## 4. Thoracic Aorta Fistulas

Pathologic communication between the thoracic aorta and esophagus or tracheobronchial tree is a rare vascular condition and most commonly develops after open or endovascular aortic repair complicated by infection. Patients with aortoesophageal or tracheobronchial fistula often present with systemic infection and are at high risk for major hemorrhage and death. Medical management is uniformly fatal.

Aortobronchial fistula (ABF) is a rare condition characterized by acute symptomatology such as intermittent or massive hemoptysis due to endobronchial bleeding. If left untreated, patients tend to have poor prognosis with a high mortality rate [115]. ABF is often associated (1.5–1.9%) with a history of aortic surgery or TEVAR due to endoleak or stent graft oversizing by ≥20 mm). Studies have shown that complications may occur many years after the intervention [24]. The mechanism of ABF is mainly owed to external compression by the enlarging aortic aneurysm and erosion [116]. The continuous compressions on the aorta by the adjacent lesions (aortic aneurysm or PSA, surgical sutures, aortic stent grafting, severe lung infection, and patent ductus arteriosus) may lead to inflammation and scar formation and eventually ABF. These fistulas usually involve the left side of the bronchial tree because of the narrow distance between the DTA and the left bronchial hemisystem being the consequence of local infection and pseudoaneurysms. The treatment of ABF can be surgical or by TEVAR [117].

Aortoesophageal fistula (AEF) is a rare cause of severe upper gastrointestinal hemorrhage with an associated mortality of 77% with treatment and 100% fatal without intervention [118]. Primary AEFs are rare but highly lethal usually caused by intraesophageal rupture of an atherosclerotic, syphilitic, or dissecting aneurysm of the DTA [118].

**Figure 13:**
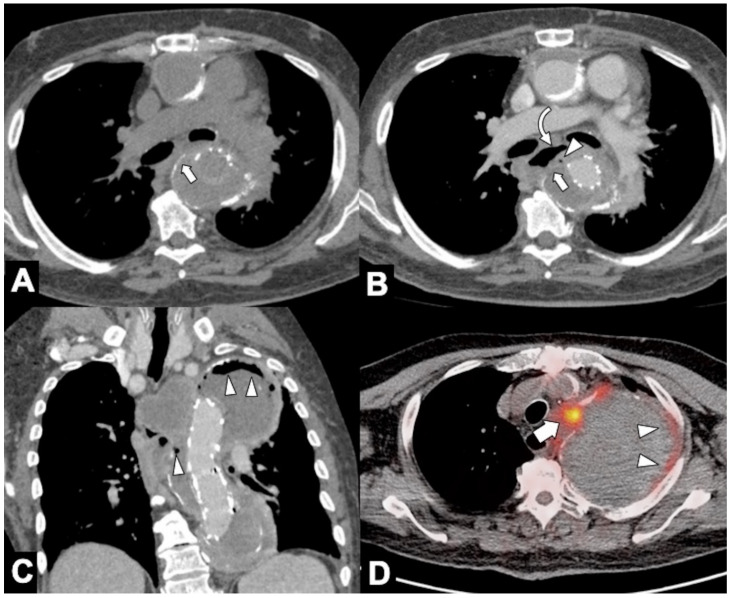
CT evaluation and CT-PET in a 68-year-old male patient with an aortoesophageal fistula (AEF) after 28 days from TEVAR. (**A**,**B**) CTA evaluation for the onset of fever showing disappearance of thoracic descending aortic wall calcification (arrow), distended esophagus (curved arrow), and appearance of an ectopic small air bubble in the native aneurysmal aortic lumen (arrowhead). (**C**) Coronal oblique MPR image better shows a significant amount of air in the native aortic lumen (arrowheads) due to probable AEF. (**D**) Fused FDG PET-CT axial image shows multiple foci of increased FDG uptake both in the aneurysm wall (arrowheads) both in the mediastinum (arrow) suggestive of infected thoracic aortic aneurysm and TEVAR endograft.

Most cases are secondary AEFs caused by a swallowed foreign body, esophageal carcinoma, cardiovascular surgery, an infected aortic graft, or erosion of an endovascular stent into the esophagus [118,119]. The classic triad of symptoms of aortoesophageal fistulas are mid-thoracic pain and a sentinel hemorrhage, followed by massive bleeding after a symptom-free interval. Such herald bleeding (self-limited) is reported in up to 50–90% of patients. In such cases, unenhanced CT studies may show abnormal aortic walls, extraluminal gas adjacent to the aortic lumen, and effacement of the periaortic fat plane; oral water-soluble contrast agents may reveal extrinsic compression or displacement of the esophagus by the aneurysm but rarely show leakage of contrast medium into the aorta because of the different flow dynamics of these structures (Figure 13). CTA may show aortic bulging but more rarely the presence of a mycotic aneurysm and signs of direct extravasation of vascular contrast or graft migration into the esophagus. The minimally invasive TEVAR approach, often in combination with esophageal intervention (endoscopic or surgical), is potentially lifesaving [119].

## 5. Pseudoaneurysms (PSA), Infectious (Mycotic), and Noninfectious Aneurysms

By definition, false aneurysms or PSA arise from a disruption in arterial wall continuity, having a wall with less than three layers since they are contained by the adventitia or merely by Peri-adventitial connective tissue. They typically have a saccular morphology with a narrow neck and are most commonly consequent to trauma, PAU, or infection (mycotic aneurysms) [120].

Inflammatory processes of the aortic layers or autoimmune processes involving the vasa vasorum supplying the aortic wall lead to weakening, stenosis, expansion, and dissection [121].

Aortitis is a general term used for a spectrum of inflammatory disorders involving the aorta. The causes of aortitis are most easily sorted into either infectious (Syphilis, Salmonella, Staphylococcus, etc.) or noninfectious (several forms of primary and secondary large-vessel vasculitis) disease [122]. Ductus diverticulum consists of a developmental convex focal outpouching in the inferior aspect of the isthmic region of the aortic arch (at the site of the ductus arteriosus), which may be mistaken for an acute aortic injury. In contrast to a PSA, ductus diverticulum has smooth and symmetric margins forming obtuse angles with the aortic wall [123].

### 5.1. Infectious Aortitis

Mycotic or infective aneurysms, currently rather rare due to prompt treatment with antibiotics, are localized and irreversible vascular dilatations caused by weakening, loss of tone, erosion, and subsequent destruction of the vessel wall by an invasive organism. Endarterial infection may arise through hematogenous seeding from distant septic foci, such as endocardial vegetations, infected thrombi, or implanted intravascular devices, with infected particles reaching the arterial intima or deeper mural layers via the vasa vasorum. Other ways of spreading of infection include the lymphatic system (particularly tuberculosis), contiguous extension such as purulent pericarditis or osteomyelitis, direct inoculation in cases of angiography, or through intravenous drug misuse.

**Figure 14:**
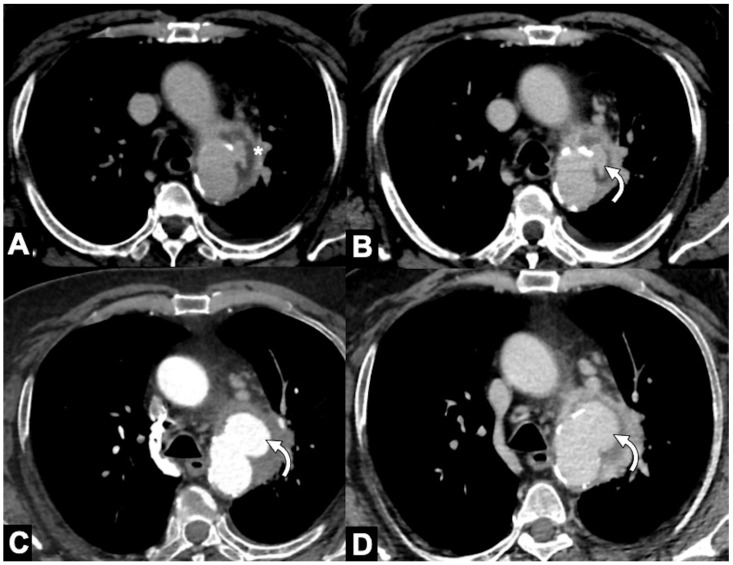
CTA of aortitis and periaortitis by Salmonella species. A 68-year-old man with chills, high temperature, and upper back pain radiating to anterior chest. (**A**) Unenhanced CTA axial image shows marginal periaortic tissue (asterisk) at aortic isthmus level. (**B**) Axial CTA late phase image also shows a focal outpouching (curved arrow) of contrast material emanating from the isthmus. (**C**,**D**) Axial arterial and delayed-phase CTA images obtained 7 days later show that contrast outpouching was considerably larger (curved arrows), a finding referable to rapidly growing focal mycotic pseudoaneurysm, and the patient underwent endovascular aortic repair and intravenous antibiotic therapy.

Mycotic aneurysms usually affect major arteries, classically at branch points, and have a propensity to involve the ascending aorta, which is in proximity to valves affected by endocarditis. The natural history of untreated mycotic aneurysms is dramatically fatal due to the high possibility of massive bleeding or rapidly fulminating sepsis [124]. Suggestive CTA features include new aneurysmal formation (usually saccular) with hazy and irregular contours, the presence of eccentric thrombus, rapid aneurysmal expansion, and morphological change in the shape of the aneurysm (Figure 14); synchronous aneurysms are not rare, with periadventitial enhancement and intramural or perivascular gas, oedema, soft tissue mass, or stranding of the periaortic fat [125].

### 5.2. Non-Infectious Aortitis

At least 20% of patients with giant cell arteritis (GCA) and 50–70% of patients with Takayasu’s arteritis (TA) will develop changes consistent with aortitis [126,127]. There is segmental and patchy granulomatous inflammation of the aorta typically resulting in arterial stenosis by medial fibrosis and occlusion; however, aneurysms may also occur due to destruction of the elastic fibers [128]. It is unclear why some cases of non-infectious aortitis lead to aneurysm formation and others to stenosis. Sometimes both entities may occur concomitantly at different anatomical areas in the same patient. CT features include high attenuation of the thickened aortic wall with calcification present on the nonenhanced scans. Arterial enhancement is considered a sign of active disease [126,127,128]. PET/CT has high sensitivity for medium- to large-vessel vasculitis and can be very useful in demonstrating aortic graft infection [129].

Notably, both aortitis and periaortitis may arise in the context of IgG4-related disease that is defined as idiopathic inflammatory and sclerosing lesions infiltrated by numerous IgG4-positive plasma cells with multi-organ effects, including the vascular system [130,131]. More common CTA findings are severe, often circumferential, aortic wall thickening with homogenous delayed-phase aortic mural enhancement and minor or major irregular extension of periaortic inflammatory tissue into adjacent adipose tissue. Luminal changes (often dilation and more rarely stenosis) may also be present [131].

## 6. Aortic Acute Occlusive Disease (And Shaggy Aorta)

Spontaneous acute thromboatheromatous embolization from diffuse aortic atherosclerotic disease is an unusual, life-threatening, and poorly understood entity that is also termed the “shaggy” aorta (SA) syndrome, since that on CT scans and angiograms the aorta has a spiculated and irregular appearance caused by irregular mural thrombi [132,133,134]. The extremely friable, severe aortic surface degeneration results in multiple recurrent microembolizations, which arise from extensive atheromatous ulcerations. SA is also associated with a high risk of diffuse embolism after catheterization, aortic manipulation, surgery, or minimally invasive procedures such as trans-catheter aortic valve implantation or TEVAR [135,136].

**Figure 15:**
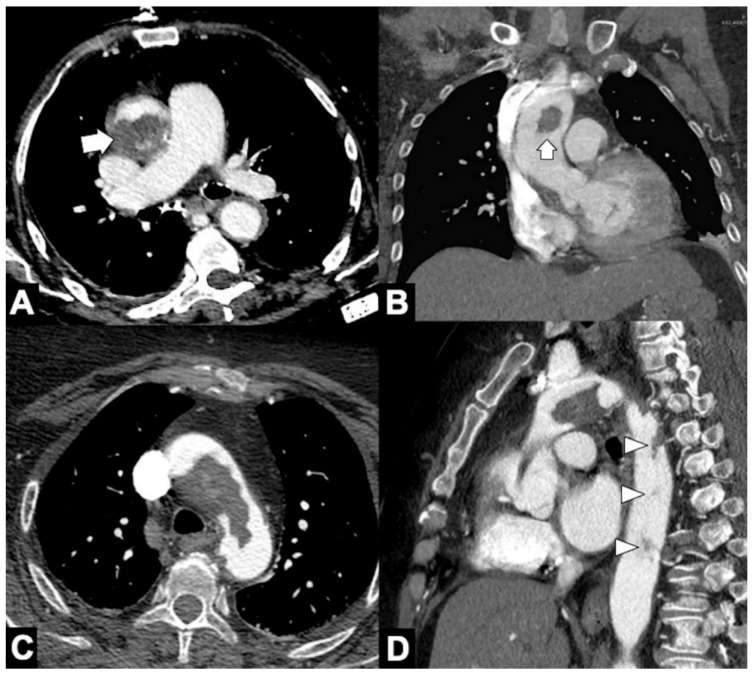
CTA of acute diffuse aortic atheromatous embolization in a 46-year-old COVID-19 pneumonia patient with acute cerebral vascular event. (**A**) CTA axial scan shows extensive and irregular atheroma of the ascending aorta (arrow). (**B**) Coronal MIP reconstruction image confirms ascending aorta filling defect (arrow). (**C**) In the same patient, CTA axial scan shows extensive irregular atheroma of the transverse aortic arch. (**D**) Sagittal MIP reconstruction image confirms extensive arch atheroma and friable floating thrombi in descending thoracic aorta (arrowheads).

Acute thrombotic involvement of the aorta, also along its thoracic course, has been recently described in SARS-CoV-2 syndrome (Figure 15) [137]. The clinical features of this syndrome depend on the level of occlusion and may include acute peripheral embolization alone (causing livido reticularis of the buttocks, thighs, legs, feet, and frequently multiple ischemic toes), visceral embolization alone (causing hematuria and acute renal failure, spinal cord injury and stroke, pancreatitis or bowel infarction), or both visceral and peripheral embolization.

To predict embolic complications, Maeda et al. developed an embolic predictor scoring system on pre-procedural (TAVI, TEVAR) CTA defining and quantifying SA. One shaggy point was given for the presence of: (1) ulcer-like thrombus, (2) maximum thrombus thickness > 5 mm, and (3) mural thrombus that occupies more than two thirds of the circumference of the aortic diameter on reconstructed CT axial images. Subsequently, each point was added to obtain the total shaggy score. The cut-off value was three points [138]. Coral Reef Aorta is a rare phenomenon characterized by the presence of internally protruding calcification involving the suprarenal or juxtarenal aorta that causes significant obstruction of the lumen [139,140]. Patients may develop complications including downstream embolic events, multiple organ failure, and even death.

## 7. Conclusions

TAE remains a challenging disease process that requires clinical vigilance, rapid diagnosis, and goal-directed management. High-quality aortic imaging plays a central role in the management of patients with TAE. While there are a wide range of potential etiologies for TAE, the majority of these diseases are managed based on morphologic findings. To ensure optimal patient care, radiologists must be familiar with potential sources of artifact and measurement error. A complete imaging report should not only describe aortic dimensions but also provide a complete description of the extent of the disease, detail morphologic features that suggest a specific TAE etiology, and emphasize secondary imaging features that imply additional risks. Finally, we recognize the urgent need to increase awareness of thoracic aorta emergencies worldwide, including dedicated education/prevention programs, and to improve diagnostic and therapeutic strategies, outcomes, and lifelong surveillance.

## Figures and Tables

**Table 1:** Non-traumatic thoracic aortic emergencies.

Acute Aortic Syndrome (AAS): Dissection and its variants
*Classic dual-barrel aortic dissection (AD)*
*Intramural hematoma without intimal tear (IMH)*
*Penetrating atherosclerotic ulcer (PAU)*
*Limited intimal tear*
Unstable and impending rupture of thoracic aorta aneurysms (TAA)
Thoracic aorta fistulas
Pseudoaneurysms (PSA) and infectious (mycotic) and noninfectious aneurysms
Acute aortic occlusive disease (and shaggy aorta)

**Table 3:** Complete list of morphologic features in AAS that need to be covered by imaging. The Ten Commandments in AAS.

1	Comparison with prior examinations, if available.
2	Visualization of intimal flap and its extent according to the aortic anatomic segmentation.
3	The aortic root including coronary artery perfusion and valve function (regurgitation!) and morphology (tricuspid versus bicuspid).
4	Site, size, and number of the entry tear(s) and all other distally appearing tears including re-entry tears and type and hemodynamic conditions of all side-branch involvement (static or dynamic flow impairment; no flow/low flow).
5	Diameter, length, course, and CT findings of the false lumen; the aortic maximum diameter, localization, and extent of aortic wall thickening; IMH co-existence.
6	Patency of all aortic side branches up to the Circle of Willis and caudad to the femoral bifurcation.
7	Angulation, tortuosity, and precise caliber measurement of all segments of the aorta and iliac arteries; presence of PAU (localization, length, and depth).
8	Morphologic or hemodynamic signs of organ malperfusion.
9	Pericardial effusion/tamponade; pleural/extrapleural effusion/hemorrhage; mediastinal hematoma.
10	Signs of contained (peri-aortic bleeding) or free rupture.

**Table 4:** CTA findings to differentiate true and false lumen.

True Lumen	False Lumen
Surrounded by calcifications (if present)	Delayed enhancement, slower flow
Hyperdense in early arterial phase	Hyperdense in the venous phase
Smaller than a false lumen	Larger than a true lumen
Continuity with an undissected aorta	Not connected to the unaffected aorta
Intima displaced inwards	Beak-sign, Cobwebs sign
Calcification along the intimal flap	Circular configuration
Outer wall calcification/s	Lack of outer wall calcification/s
Usually origin of CT, SMA, and RRA ^1^	Usually origin of LRA ^2^
Inner lumen in aortic arch	Partial thrombus formation
Wrapped around the false lumen	Wrapped around the true lumen

^1^ CT, celiac tripod; SMA, superior mesenteric artery; RRA, right renal artery; ^2^ LRA, left renal artery.

**Table 5:** MDCT and clinical features suggesting risk of progression of IMH.

› Ascending aorta involved (type A IMH).
› Aortic diameter > 5 cm (a greater stress on the dilated aortic wall implies a greater risk of rupture).
› Hematoma thickness (HT) > 11 mm.
› Luminal compression ratio (minimum/maximum transverse luminal diameters at the site of the maximal HT).
› Associated penetrating atherosclerotic ulcer (PAU) diameter > 20 mm and depth > 10 mm.
› Temporal aortic enlargement on serial imaging (rapid aortic diameter growth during hospital stay).
› Periaortic, pleural, or pericardial effusions, particularly if large or temporally progressive.
› Persistent pain or hemodynamic instability, or both.

**Table 6:** Classification of signs of impending and complete aortic aneurysm rupture according to location (intramural, luminal, and extraluminal).

Location	CTA Findings	Complete Rupture	Impending Rupture
Intramural	Increased aneurysm (>5.5 cm)	-	+
	Rapid enlargement rate (>4 mm/year)	-	+
	Focal wall irregularity	+	+
	Hyperattenuating crescent sign	-	+
	Thrombus fissuration	-	+
	Draped Aorta sign	-	+
	Missing calcium sign	-	+
	Tangential calcium sign	-	+
Luminal	Aortoesophageal fistula	+	-
	Aortobronchial fistula	+	-
	Periaortic stranding	-	+
Extraluminal	Contrast extravasation	+	-
	Mediastinal hematoma	+	-
	Pleural hematoma	+	-
	Pericardial hematoma	+	±

## Data Availability

The data are available from the corresponding author, T.V., upon reasonable request.

## Abbreviations

AAS, acute aortic syndromes; AD, aortic dissection; ADD-RS, aortic dissection detection risk score; BCT, brachiocephalic trunk; CM, contrast material; CTA, multidetector CT angiography; DD, D-dimer; DTA, descending thoracic aorta; ED, emergency department; FDG, fluorodeoxyglucose; FL, false lumen; IBP, intramural blood pool; IMH, acute intramural hematoma; IRAD, international registry of acute aortic dissection; LIT, limited intimal tear; LSA, left subclavian artery; MIP, maximum intensity projection; MPR, multiplanar reconstruction; PAU, penetrating atherosclerotic ulcer; PET, positron emission tomography; PSA, pseudoaneurysm; SA, shaggy aorta; SIR, signs of impending ruptures; STJ, sinotubular junction; TA, Takayasu’s arteritis; TAA, thoracic aorta aneurysm; TAAD, type A aortic dissection; TAE: thoracic aorta emergencies; TBAD, type B aortic dissection; TEVAR, thoracic endovascular aortic repair; TL, true lumen; ULP, ulcer-like projection; VR, volume rendering.

## References

[1] Jen J.P., Malik A., Lewis G., Holloway B. (2020). Non-traumatic thoracic aortic emergencies: Imaging diagnosis and management. Br. J. Hosp. Med..

[2] Voitle E., Hofmann W., Cejna M. (2015). Aortic emergencies-diagnosis and treatment: A pictorial review. Insights Imaging.

[3] Lai V., Tsang W.K., Chan W.C., Yeung T.W. (2012). Diagnostic accuracy of mediastinal width measurement on posteroanterior and anteroposterior chest radiographs in the depiction of acute nontraumatic thoracic aortic dissection. Emerg. Radiol..

[4] von Kodolitsch Y., Nienaber C.A., Dieckmann C., Schwartz A.G., Hofmann T., Brekenfeld C. (2004). Chest radiography for the diagnosis of acute aortic syndrome. Am. J. Med..

[5] Chawla A., Rajendran S., Yung W.H., Babu S.B., Peh W.C. (2016). Chest radiography in acute aortic syndrome: Pearls and pitfalls. Emerg. Radiol..

[6] Hagan P.G., Nienaber C.A., Isselbacher E.M., Bruckman D., Karavite D.J., Russman P.L., Evangelista A., Fattori R., Suzuki T., Oh J.K. (2000). The International Registry of Acute Aortic Dissection (IRAD): New insights into an old disease. JAMA.

[7] Fleischmann D., Mitchell R.S., Miller D.C. (2009). Acute Aortic Syndromes: New Insights from Electrocardiographically Gated Computed Tomography. Semin. Thorac. Cardiovasc. Surg..

[8] Sathiadoss P., Haroon M., Wongwaisayawan S., Krishna S., Sheikh A.M. (2020). Multidetector Computed Tomography in Traumatic and Nontraumatic Aortic Emergencies: Emphasis on Acute Aortic Syndromes. Can. Assoc. Radiol. J..

[9] Morello F., Santoro M., Fargion A.T., Grifoni S., Nazerian P. (2020). Diagnosis and management of acute aortic syndromes in the emergency department. Intern. Emerg. Med..

[10] Zamorano J.L., De Isla L.P., González R., Almería C., Rodrigo J.L. (2003). Imaging diagnosis in acute aortic syndromes. Rev. Esp. Cardiol..

[11] Murillo H., Lane M.J., Punn R., Fleischmann D., Restrepo C.S. (2012). Imaging of the Aorta: Embryology and Anatomy. Semin. Ultrasound CT MRI.

[12] Mei C.C., Zhang J., Jing H.X. (2018). Fluid mechanics of Windkessel effect. Med. Biol. Eng. Comput..

[13] Elefteriades J.A. (2008). Thoracic Aortic Aneurysm: Reading the Enemy’s Playbook. Yale J. Boil. Med..

[14] Ishimaru S. (2004). Endografting of the aortic arch. J. Endovasc. Ther..

[15] Criado F.J. (2010). Mapping the Aorta: A New Look at Vascular Anatomy in the Era of Endograft Repair. J. Endovasc. Ther..

[16] Hutchinson S.J. (2009). Aortic Physiology and Function: Anatomic and Histologic Considerations. Aortic Diseases Clinical Diagnostic Imaging Atlas.

[17] Ko J.P., Goldstein J.M., Latson L.A., Azour L., Gozansky E.K., Moore W., Patel S., Hutchinson B., Chest C.T. (2021). Angiography for Acute Aortic Pathologic Conditions: Pearls and Pitfalls. RadioGraphics.

[18] Kalisz K., Halliburton S., Abbara S., Leipsic J.A., Albrecht M.H., Schoepf U.J., Rajiah P. (2017). Update on Cardiovascular Applica-tions of Multienergy CT. RadioGraphics.

[19] Smettei O.A., Sayed S., Al Habib A.M., Alharbi F., Abazid R.M. (2018). Ultra-fast, low dose high-pitch (FLASH) versus prospectively-gated coronary computed tomography angiography: Comparison of image quality and patient radiation exposure. J. Saudi Heart Assoc..

[20] Rajiah P. (2020). Updates in Vascular Computed Tomography. Radiol. Clin. N. Am..

[21] Baliyan V., Shaqdan K., Hedgire S., Ghoshhajra B. (2019). Vascular computed tomography angiography technique and indications. Cardiovasc. Diagn. Ther..

[22] Valente T., Rossi G., Lassandro F., Rea G., Marino M., Muto M., Molino A., Scaglione M. (2016). MDCT evaluation of acute aortic syndrome (AAS). Br. J. Radiol..

[23] Vilacosta I., Roman J.A. (2001). Acute aortic syndrome. Heart.

[24] Erbel R., Aboyans V., Boileau C., Bossone E., Bartolomeo R.D., Eggebrecht H., Evangelista A., Falk V., Frank H., Gaemperli O. (2014). 2014 ESC Guidelines on the diagnosis and treatment of aortic diseases: Document covering acute and chronic aortic diseases of the thoracic and abdominal aorta of the adult. The task force for the diagnosis and treatment of Aortic diseases of the European Society of Cardiology (ESC). Eur. Heart J..

[25] Hiratzka L.F., Bakris G.L., Beckman J.A., Bersin R.M., Carr V.F., Casey D.E., Eagle K.A., Hermann L.K., Isselbacher E.M., Kazerooni E.A. (2010). ACCF/AHA/AATS/ACR/ASA/SCA/SCAI/SIR/STS/SVM guidelines for the diagnosis and management of patients with Thoracic Aortic Disease: A report of the American College of Cardiology Foundation/American Heart Association Task Force on Practice Guidelines, American Association for Thoracic Surgery, American College of Radiology, American Stroke Association, Society of Cardiovascular Anesthesiologists, Society for Cardiovascular Angiography and Interventions, Society of Interventional Radiology, Society of Thoracic Surgeons, and Society for Vascular Medicine. Circulation.

[26] Svensson L.G., Labib S.B., Eisenhauer A.C., Butterly J.R. (1999). Intimal tear without hematoma: An important variant of aortic dissection that can elude current imaging techniques. Circulation.

[27] Grist T.M., Rubin G.D., Hodler J., Kubik-Huch R.A., von Schulthess G.K. Imaging of Acute Aortic Syndromes. Diseases of the Chest, Breast, Heart and Vessels 2019–2022.

[28] Nazerian P., Mueller C., de Matos Soeiro A., Leidel B.A., Salvadeo S.A.T., Giachino F., Vanni S., Grimm K., Oliveira M.T., Pivetta E. (2018). Diagnostic Accuracy of the Aortic Dissection Detection Risk Score Plus D-Dimer for Acute Aortic Syndromes: The ADvISED Prospective Multicenter Study. Circulation.

[29] Tsutsumi Y., Tsujimoto Y., Takahashi S., Tsuchiya A., Fukuma S., Yamamoto Y., Fukuhara S. (2020). Accuracy of aortic dissection detection risk score alone or with D-dimer: A systematic review and meta-analysis. Eur. Hear. J. Acute Cardiovasc. Care.

[30] Kelly A.-M. (2021). Why the aortic dissection detection risk score is problematic in emergency departments. Explor. Med..

[31] Myrmel T., Larsen M., Bartnes K. (2016). The International Registry of Acute Aortic Dissections (IRAD)—Experiences from the first 20 years, Scand. Cardiovasc. J..

[32] Pepper J. (2016). Differential aspects of the disease and treatment of thoracic acute aortic dissection (TAAD)—The European experience. Ann. Cardiothorac. Surg..

[33] Rylski B., Pacini D., Beyersdorf F., Quintana E., Schachner T., Tsagakis K., Ronchey S., Durko A., De Paulis R., Siepe M. (2019). Standards of reporting in open and endovascular aortic surgery (STORAGE guidelines). Eur. J. Cardio-Thoracic Surg..

[34] Kapoor V., Ferris J.V., Fuhrman C.R. (2004). Intimomedial Rupture: A New CT Finding to Distinguish True from False Lumen in Aortic Dissection. Am. J. Roentgenol..

[35] Castañer E., Andreu M., Gallardo X., Mata J.M., Cabezuelo M.A., Pallardo Y. (2003). CT in nontraumatic acute thoracic aortic disease: Typical and atypical features and complications. Radiographics.

[36] McMahon M.A., Squirrell C.A. (2010). Multidetector CT of Aortic Dissection: A Pictorial Review. RadioGraphics.

[37] Maddu K.K., Shuaib W., Tellería J., Johnson J.-O., Khosa F. (2014). Nontraumatic Acute Aortic Emergencies: Part 1, Acute Aortic Syndrome. Am. J. Roentgenol..

[38] Litmanovich D., Bankier A.A., Cantin L., Raptopoulos V., Boiselle P.M. (2009). CT and MRI in Diseases of the Aorta. Am. J. Roentgenol..

[39] LePage M.A., Quint L.E., Sonnad S.S., Deeb G.M., Williams D.M. (2001). Aortic dissection: CT features that distinguish true lumen from false lumen. AJR Am. J. Roentgenol..

[40] Moullet P., Mann H. (2014). Beak sign. J. Thorac. Imaging.

[41] Han J., Xiang H., Ridley W.E., Ridley L.J. (2018). Aortic webs and cobwebs: Aortic dissection and arteriopathies. J. Med. Imaging Radiat. Oncol..

[42] Qanadli S.D., Malekzadeh S., Villard N., Jouannic A.-M., Bodenmann D., Tozzi P., Rotzinger D.C. (2020). A New Clinically Driven Classification for Acute Aortic Dissection. Front. Surg..

[43] de Farias L.P.G., Santos J.M.M.M., Teles G.B.D.S., Baptista L.P.S. (2020). Intimointimal Intussusception in Acute Aortic Dissection. Radiol. Cardiothorac. Imaging.

[44] Shin M.S., Zorn G.L., Ho K.J. (1988). Computed tomography manifestation of a triple-barreled aortic dissection: The Mercedes-Benz mark sign. J. Comput. Tomogr..

[45] Daily P.O., Trueblood H.W., Stinson E.B., Wuerflein R.D., Shumway N.E. (1970). Management of Acute Aortic Dissections. Ann. Thorac. Surg..

[46] DeBakey M.E., Henly W.S., Cooley D.A., Morris G.C., Crawford E.S., Beall A.C. (1965). Surgical management of dissecting aneurysm of the aorta. J. Thorac. Cardiovasc. Surg..

[47] Rylski B., Pérez M., Beyersdorf F., Reser D., Kari F.A., Siepe M., Czerny M. (2017). Acute non-A non-B aortic dissection: Incidence, treatment and outcome. Eur. J. Cardio-Thoracic Surg..

[48] Carino D., Singh M., Molardi A., Agostinelli A., Goldoni M., Pacini D., Nicolini F. (2019). Non-A non-B aortic dissection: A systematic review and meta-analysis. Eur. J. Cardiothorac. Surg..

[49] Sievers H.-H., Rylski B., Czerny M., Baier A.L.M., Kreibich M., Siepe M., Beyersdorf F. (2019). Aortic dissection reconsidered: Type, entry site, malperfusion classification adding clarity and enabling outcome prediction. Interact. Cardiovasc. Thorac. Surg..

[50] Evangelista A., Isselbacher E.M., Bossone E., Gleason T.G., DI Eusanio M., Sechtem U., Ehrlich M.P., Trimarchi S., Braverman A.C., Myrmel T. (2018). Insights From the International Registry of Acute Aortic Dissection. Circulation.

[51] Rampoldi V., Trimarchi S., Eagle K.A., Nienaber C.A., Oh J.K., Bossone E., Myrmel T., Sangiorgi G., De Vincentiis C., Cooper J.V. (2007). Simple Risk Models to Predict Surgical Mortality in Acute Type A Aortic Dissection: The International Registry of Acute Aortic Dissection Score. Ann. Thorac. Surg..

[52] Nauta F.J., Tolenaar J.L., Patel H.J., Appoo J.J., Tsai T.T., Desai N.D., Montgomery D.G., Mussa F.F., Upchurch G.R., Fattori R. (2016). Impact of Retrograde Arch Extension in Acute Type B Aortic Dissection on Management and Outcomes. Ann. Thorac. Surg..

[53] Marchand P. (1951). The Anatomy and Applied Anatomy of the Mediastinal Fascia. Thorax.

[54] Panicek D.M., Ewing D.K., Markarian B., Heitzman E.R. (1987). Interstitial pulmonary hemorrhage from mediastinal hematoma secondary to aortic rupture. Radiology.

[55] Sueyoshi E., Sakamoto I., Uetani M., Matsuoka Y., Suenaga E. (2007). CT Findings of Ruptured Intramural Hematoma of the Aorta Extending Along the Pulmonary Artery. Cardiovasc. Interv. Radiol..

[56] Sueyoshi E., Matsuoka Y., Sakamoto I., Uetani M. (2009). CT and clinical features of hemorrhage extending along the pulmonary artery due to ruptured aortic dissection. Eur. Radiol..

[57] Cao D.B., Yang S.R., Tong Q., Zheng Y. (2010). Interstitial pulmonary hemorrhage along the pulmonary artery secondary to ruptured aortic dissection. Intern. Med..

[58] Guilmette J., Semionov A., Dennie C., Gahide G., Pressacco J., Fraser R., Cordeau M.-P., Chartrand-Lefebvre C. (2016). Hemorrhagic infiltration of the aortopulmonary adventitia: A complication of acute aortic dissection. Eur. J. Radiol..

[59] Lee K.-C., Lee J.W., Yong H.S., Kang E.-Y. (2018). Different CT Findings of Aortic Hemorrhage Extending to Pulmonary Artery from Stanford Type A Aortic Dissection. Iran. J. Radiol..

[60] de Farias L.P.G., Favaretto A.C., Baptista L.P.S., Teles G.B.S. (2021). Pulmonary Arterial Intramural Hematoma Due to Acute Aortic Dissection. Arq. Bras. Cardiol..

[61] Fattori R., Tsai T.T., Myrmel T., Evangelista A., Cooper J.V., Trimarchi S., Li J., Lovato L., Kische S., Eagle K.A. (2008). Complicated Acute Type B Dissection: Is Surgery Still the Best Option? A Report From the International Registry of Acute Aortic Dissection. JACC Cardiovasc. Interv..

[62] Ray H.M., Durham C.A., Ocazionez D., Charlton-Ouw K.M., Estrera A.L., Miller C.C., Safi H.J., Azizzadeh A. (2016). Predictors of intervention and mortality in patients with uncomplicated acute type B aortic dissection. J. Vasc. Surg..

[63] Fattori R., Cao P., De Rango P., Czerny M., Evangelista A., Nienaber C., Rousseau H., Schepens M. (2013). Interdisciplinary Expert Consensus Document on Management of Type B Aortic Dissection. J. Am. Coll. Cardiol..

[64] Luebke T., Brunkwall J. (2014). Type B Aortic Dissection: A Review of Prognostic Factors and Meta-analysis of Treatment Options. Aorta.

[65] Nauta F.J., Trimarchi S., Kamman A.V., Moll F.L., van Herwaarden J.A., Patel H.J., Figueroa C.A., Eagle K.A., Froehlich J.B. (2016). Up-date in the management of type B aortic dissection. Vasc. Med..

[66] Kaji S. (2018). Update on the Therapeutic Strategy of Type B Aortic Dissection. J. Atheroscler. Thromb..

[67] Tsai T.T., Isselbacher E.M., Trimarchi S., Bossone E., Pape L., Januzzi J.L., Evangelista A., Oh J.K., Llovet A., Beckman J. (2007). Acute type B aortic dissection: Does aortic arch involvement affect management and outcomes? Insights from the International Registry of Acute Aortic Dissection (IRAD). Circulation.

[68] Valentine R.J., Boll J.M., Hocking K., Curci J.A., Garrard C.L., Brophy C.M., Naslund T.C. (2016). Aortic arch involvement worsens the prognosis of type B aortic dissections. J. Vasc. Surg..

[69] Czerny M., Schmidli J., Adler S., van den Berg J.C., Bertoglio L., Carrel T., Chiesa R., Clough R.E., Eberle B., Etz C. (2019). Editor’s Choice—Current Options and Recommendations for the Treatment of Thoracic Aortic Pathologies Involving the Aortic Arch: An Expert Consensus Document of the European Association for Cardio-Thoracic Surgery (EACTS) & the European Society for Vascular Surgery (ESVS). Eur. J. Vasc. Endovasc. Surg..

[70] Nienaber C.A., Sievers H.H. (2002). Intramural hematoma in acute aortic syndrome—More than one variant of dissection?. Circulation.

[71] Ganaha F., Craig Miller D., Sugimoto K., Do Y.S., Minamiguchi H.M.D., Saito H., Scott Mitchell R., Dake M.D. (2002). Prognosis of Aortic Intramural Hematoma With and Without Penetrating Atherosclerotic Ulcer. Circulation.

[72] Harris K.M., Braverman A.C., Eagle K.A., Woznicki E.M., Pyeritz R.E., Myrmel T., Peterson M.D., Voehringer M., Fattori R., Januzzi J.L. (2012). Acute aortic intramural hematoma: An analysis from the International Registry of Acute Aortic Dissection. Circulation.

[73] Krukenberg E. (1920). Beitrage zur Frage des Aneurisms dissecans. Beitr. Pathol. Anat. Allg. Pathol..

[74] Kitai T., Kaji S., Yamamuro A., Tani T., Kinoshita M., Ehara N., Kobori A., Kim K., Kita T., Furukawa Y. (2011). Detection of Intimal Defect by 64-Row Multidetector Computed Tomography in Patients With Acute Aortic Intramural Hematoma. Circulation.

[75] Mussa F.F., Horton J.D., Moridzadeh R., Nicholson J., Trimarchi S., Eagle K.A. (2016). Acute aortic dissection and intramural hematoma: A systematic review. JAMA.

[76] Maslow A., Atalay M.K., Sodha N. (2018). Intramural Hematoma. J. Cardiothorac. Vasc. Anesth..

[77] Sueyoshi E., Matsuoka Y., Sakamoto I., Uetani M., Hayashi K., Narimatsu M. (1997). Fate of Intramural Hematoma of the Aorta: CT Evaluation. J. Comput. Assist. Tomogr..

[78] Lee Y.K., Seo J.B., Jang Y.M., Do K.H., Kim S.S., Lee J.S., Song K.S., Song J.W., Han H., Kim S.S. (2007). Acute and chronic complications of aortic intramural hematoma on follow-up computed tomography: Incidence and predictor analysis. J. Comput. Assist. Tomogr..

[79] Ferrera C., Vilacosta I., Cabeza B., Cobiella J., Martínez I., Sanz M.S.-P., Bustos A., Serrano F.J., Maroto L. (2020). Diagnosing Aortic Intramural Hematoma: Current Perspectives. Vasc. Health Risk Manag..

[80] Oderich G.S., Kärkkäinen J.M., Reed N.R., Tenorio E., Sandri G.A. (2018). Penetrating Aortic Ulcer and Intramural Hematoma. Cardiovasc. Interv. Radiol..

[81] Murillo H., Molvin L.A., Chin S., Fleischmann D. (2021). Aortic Dissection and Other Acute Aortic Syndromes: Diagnostic Imaging Findings from Acute to Chronic Longitudinal Progression. RadioGraphics.

[82] Evangelista A., Dominguez R., Sebastia C., Salas A., Permanyer-Miralda G., Avegliano G., Gomez-Bosh Z., Gonzalez-Alujas T., Garcia del Castillo H., Soler-Soler J. (2004). Prognostic value of clinical and morphologic findings in short-term evolution of aortic intramural hematoma. Eur. Heart J..

[83] Vilacosta I., Román J.A.S., Ferreirós J., Aragoncillo P., Méndez R., Castillo J.A., Rollán M.J., Batlle E., Peral V., Sánchez-Harguindey L. (1997). Natural history and serial morphology of aortic intramural hematoma: A novel variant of aortic dissection. Am. Heart J..

[84] Schlatter T., Auriol J., Marcheix B., Lebbadi M., Marachet M.A., Dang-Tran K.D., Tran M., Honton B., Gardette V., Rousseau H. (2011). Type B Intramural Hematoma of the Aorta: Evolution and Prognostic Value of Intimal Erosion. J. Vasc. Interv. Radiol..

[85] Kitai T., Kaji S., Yamamuro A., Tani T., Kinoshita M., Ehara N., Kobori A., Kita T., Furukawa Y. (2010). Impact of New Development of Ulcer-Like Projection on Clinical Outcomes in Patients With Type B Aortic Dissection With Closed and Thrombosed False Lumen. Circulation.

[86] Sueyoshi E., Matsuoka Y., Imada T., Okimoto T., Sakamoto I., Hayashi K. (2002). New Development of an Ulcerlike Projection in Aortic Intramural Hematoma: CT Evaluation. Radiology.

[87] Evangelista A., Moral S. (2020). Penetrating atherosclerotic ulcer. Curr. Opin. Cardiol..

[88] Nathan D.P., Boonn W., Lai E., Wang G.J., Desai N., Woo E.Y., Fairman R.M., Jackson B.M. (2012). Presentation, complications, and natural history of penetrating atherosclerotic ulcer disease. J. Vasc. Surg..

[89] Bansal S., Singhania N., Yadav M., Singhania G. (2020). A Penetrating Aortic Ulcer. J. Emerg. Med..

[90] Roldan C.J. (2012). Penetrating atherosclerotic ulcerative disease of the aorta: Do emergency physicians need to worry?. J. Emerg. Med..

[91] El Hassani I., Van Damme H., Creemers E., Boesmans E., Defraigne J.O. (2017). Penetrating atherosclerosis aortic ulcer: A re-appraisal. Acta Chir. Belg..

[92] Svensson L.G. (2018). Limited Intimal Aorta Tears. J. Am. Coll. Cardiol..

[93] Chirillo F., Salvador L., Bacchion F., Grisolia E.F., Valfrè C., Olivari Z. (2007). Clinical and Anatomical Characteristics of Subtle-Discrete Dissection of the Ascending Aorta. Am. J. Cardiol..

[94] La Canna G., Formisano T., Monti L., Torracca L., Scarfò I. (2019). A Subtle Clinical Phenotype of Aortic Limited Intimal Tear without Hematoma. JACC Cardiovasc. Imaging.

[95] Ruiz Carazo E., Láinez Ramos-Bossini A.J., Pérez García C., López Milena G. (2020). Aortic dissection class 3: A little-known entity. Presentation of 4 cases. Radiologia.

[96] Ueda T., Chin A., Petrovitch I., Fleischmann D. (2012). A pictorial review of acute aortic syndrome: Discriminating and overlapping features as revealed by ECG-gated multidetector-row CT angiography. Insights Imaging.

[97] Chin A.S., Willemink M.J., Kino A., Hinostroza V., Sailer A.M., Fischbein M.P., Mitchell R.S., Berry G.J., Miller D.C., Fleischmann D. (2018). Acute Limited Intimal Tears of the Thoracic Aorta. J. Am. Coll. Cardiol..

[98] Valente T., Rossi G., Lassandro F., Rea G., Marino M., Urciuolo S., Tortora G., Muto M. (2015). MDCT distinguishing features of focal aortic projections (FAP) in acute clinical settings. Radiol. Med..

[99] Gouveia E., Melo R., Silva Duarte G., Lopes A., Alves M., Caldeira D., Fernandes E., Fernandes R., Mendes Pedro L. (2021). Incidence and prevalence of thoracic aortic aneurysms: A systematic review and meta- analysis of population-based studies. Semin. Thorac. Cardiovasc. Surg..

[100] Mao S.S., Ahmadi N., Shah B., Beckmann D., Chen A., Ngo L., Flores F.R., Gao Y.L., Budoff M.J. (2008). Normal thoracic aorta diameter on cardiac computed tomography in healthy asymptomatic adults. Acad. Radiol..

[101] Elefteriades J.A., Mukherjee S.K., Mojibian H. (2020). Discrepancies in measurement of the thoracic aorta: JACC review topic of the week. J. Am. Coll. Cardiol..

[102] Obel L.M., Diederichsen A.C., Steffensen F.H., Frost L., Lambrechtsen J., Busk M., Urbonaviciene G., Egstrup K., Karon M., Rasmussen L.M. (2021). Population-Based Risk Factors for Ascending, Arch, Descending, and Abdominal Aortic Dilations for 60-74-Year-Old Individuals. J. Am. Coll. Cardiol..

[103] Gaudry M., Barral P.-A., Blanchard A., Palazzolo S., Bolomey S., Omnes V., De Masi M., Carcopino-Tusoli M., Meyrignac O., Rousseau H. (2021). Prevalence of Thoracic Aortic Aneurysms in Patients with Degenerative Abdominal Aortic Aneurysms: Results from the Prospective ACTA Study. Eur. J. Vasc. Endovasc. Surg..

[104] Safi H.J., Miller C.C. (1999). Spinal cord protection in descending thoracic and thoracoabdominal aortic repair. Ann. Thorac. Surg..

[105] Boules T.N., Compton C.N., Stanziale S.F., Sheehan M.K., Dillavou E.D., Gupta N., Tzeng E., Makaroun M.S. (2006). Can computed tomography scan findings predict ‘‘impending’’ aneurysm rupture?. Vasc. Endovasc. Surg..

[106] Schwartz S.A., Taljanovic M.S., Smyth S., O’Brien M.J., Rogers L.F. (2007). CT findings of rupture, impending rupture, and contained rupture of abdominal aortic aneurysms. AJR Am. J. Roentgenol..

[107] Kumar Y., Hooda K., Li S., Goyal P., Gupta N., Adeb M. (2017). Abdominal aortic aneurysm: Pictorial review of common appearances and complications. Ann. Transl. Med..

[108] Gish D.S., Baer J.A., Crabtree G.S., Shaikh B., Fareedy S.B. (2016). Impending aortic aneurysm rupture—A case report and review of the warning signs. J. Community Hosp. Intern. Med. Perspect..

[109] Kang E.-J., Lee K.-N., Lee J. (2017). Acute Aortic Syndrome: Recent Trends in Imaging Assessment Using Computed Tomography Angiograph. Cardiovasc. Imaging Asia.

[110] Rakita D., Newatia A., Hines J.J., Siegel D.N., Friedman B. (2007). Spectrum of CT Findings in Rupture and Impending Rupture of Abdominal Aortic Aneurysms. RadioGraphics.

[111] Roy J., Labruto F., Beckman M.O., Danielson J., Johansson G., Swedenborg J. (2008). Bleeding into the intraluminal thrombus in abdominal aortic aneurysms is associated with rupture. J. Vasc. Surg..

[112] Antunes B.F.F., Tachibana A., de Almeida Mendes C., Lembrança L., Silva M.J., Teivelis M.P., Wolosker N. (2021). Signs of impending rupture in abdominal aortic and iliac artery aneurysms by computed tomography: Outcomes in 41 patients. Clinics.

[113] Corrêa I.B., Alves B.L.T., Sobrinho T.A.D.O., Ramos L.F.M., Diniz R.L.F.C., Ribeiro M.A. (2019). Abdominal aortic aneurysms that have ruptured or are at imminent risk of rupture. Radiol. Bras..

[114] Ueda T., Hayashi H., Ando T., Iwata K., Saito H., Kumita S.I. (2021). Computed Tomography Attenuation Values of the High-Attenuating Crescent Sign Can Discriminate Between Rupture, Impending Rupture, and Non-Rupture of Aortic Aneurysms. Circ. J..

[115] Yuan S.M. (2020). Aortobronchial fistula. Gen. Thorac. Cardiovasc. Surg..

[116] Hui D.S., Stoeckel D.A., Kaufman E.E., Jacobs D.L. (2016). Massive Hemoptysis From an Aortobronchial Fistula Secondary to BCG-Related Mycotic Thoracic Aortic Aneurysm. Ann. Thorac. Surg..

[117] Tigkiropoulos K., Stavridis K., Lazaridis I., Saratzis N. (2017). Endovascular repair of aortobronchial fistula due to saccular aneurysm of thoracic aorta. Case Rep. Vasc. Med..

[118] Yang Y., Hu D., Peng D. (2018). Primary aortoesophageal fistula: A fatal outcome. Am. J. Emerg. Med..

[119] Andrade L.C., Felix-Morais R., Gil-Agostinho A., Caseiro-Alves F. (2014). Aorto-oesophageal fistula treated with emergent thoracic endovascular repair. BMJ Case Rep..

[120] Agarwal P.P., Chughtai A., Matzinger F.R.K., Kazerooni E.A. (2009). Multidetector CT of Thoracic Aortic Aneurysms. RadioGraphics.

[121] Chao C.P., Walker T.G., Kalva S.P. (2009). Natural History and CT Appearances of Aortic Intramural Hematoma. RadioGraphics.

[122] Hartlage G.R., Palios J., Barron B.J., Stillman A.E., Bossone E., Clements S.D., Lerakis S. (2014). Multimodality Imaging of Aortitis. JACC Cardiovasc. Imaging.

[123] Grollman J.H. (1995). Aortic or ductus diverticulum?. AJR Am. J. Roentgenol..

[124] Katabathina V.S., Restrepo C.S. (2012). Infectious and Noninfectious Aortitis: Cross-Sectional Imaging Findings. Semin. Ultrasound CT MRI.

[125] Murphy D.J., Keraliya A.R., Agrawal M.D., Aghayev A., Steigner M.L. (2016). Cross-sectional imaging of aortic infections. Insights Imaging.

[126] Holm P.W., Sandovici M., Slart R.H., Glaudemans A., Rutgers A., Brouwer E. (2016). Vessel involvement in giant cell arteritis: An imaging approach. J. Cardiovasc. Surg..

[127] Weinrich J.M., Lenz A., Adam G., François C.J., Bannas P. (2020). Radiologic Imaging in Large and Medium Vessel Vasculitis. Radiol. Clin. N. Am..

[128] Kang E.J., Kim S.M., Choe Y.H., Lee G.Y., Lee K.N., Kim D.K. (2014). Takayasu arteritis: Assessment of coronary arterial abnormalities with 128-section dual-source CT angiography of the coronary arteries and aorta. Radiology.

[129] Husmann L., Huellner M.W., Ledergerber B., Eberhard N., Kaelin M.B., Anagnostopoulos A., Kudura K., Burger I.A., Mestres C.-A., Rancic Z. (2020). Diagnostic Accuracy of PET/CT and Contrast Enhanced CT in Patients With Suspected Infected Aortic Aneurysms. Eur. J. Vasc. Endovasc. Surg..

[130] Stone J.R., Bruneval P., Angelini A., Bartoloni G., Basso C., Batoroeva L., Buja L.M., Butany J., d’Amati G., Fallon J.T. (2015). Consensus statement on surgical pathology of the aorta from the Society for Cardiovascular Pathology and the Association for European Cardiovascular Pathology: I. Inflammatory diseases. Cardiovasc. Pathol..

[131] Pérez-García C.N., Olmos C., Vivas D., Ferrera C., García-Arribas D., Enríquez-Vázquez D., Carnero-Alcázar M., Maroto L., Candil A.O., Sanz M.S.-P. (2019). IgG4-aortitis among thoracic aortic aneurysms. Heart.

[132] Hollier L.H., Kazmier F.J., Ochsner J., Bowen J.C., Procter C.D. (1991). “Shaggy” Aorta Syndrome with Atheromatous Embolization to Visceral Vessels. Ann. Vasc. Surg..

[133] Fukuda I., Daitoku K., Minakawa M., Fukuda W. (2013). Shaggy and calcified aorta: Surgical implications. Gen. Thorac. Cardiovasc. Surg..

[134] Serra R., Bracale U.M., Jiritano F., Ielapi N., Licastro N., Provenzano M., Andreucci M., Pingitore A., de Franciscis S., Mastroroberto P. (2020). The Shaggy Aorta Syndrome: An Updated Review. Ann. Vasc. Surg..

[135] Patel S.D., Constantinou J., Hamilton H., Davis M., Ivancev K. (2014). Editor’s choice- A shaggy aorta is associated with mesenteric embolization in patients undergoing fenestrated endografts to treat paravisceral aortic aneurysms. Eur. J. Vasc. Endovasc. Surg..

[136] Kwon H., Han Y., Noh M., Gwon J.G., Cho Y.P., Kwon T.W. (2016). Impact of shaggy aorta in patients with abdominal aortic aneurysm following open or endovascular aneurysm repair. Eur. J. Vasc. Endovasc. Surg..

[137] Gomez-Arbelaez D., Ibarra-Sanchez G., Garcia-Gutierrez A., Comanges-Yeboles A., Ansuategui-Vicente M., Gonzalez-Fajardo J.A. (2020). COVID-19-Related Aortic Thrombosis: A Report of Four Cases. Ann. Vasc. Surg..

[138] Maeda K., Ohki T., Kanaoka Y., Shukuzawa K., Baba T., Momose M. (2020). A Novel Shaggy Aorta Scoring System to Predict Embolic Complications Following Thoracic Endovascular Aneurysm Repair. Eur. J. Vasc. Endovasc. Surg..

[139] Belczak S.Q., Sincos I.R., Aun R., Costa K.V., Araujo E.A. (2014). Coral reef aorta, emergency surgical: Case report and literature review. Einstein.

[140] Kloth C., Schmidt S.A. (2019). Coral-reef-aorta. Abdom. Radiol..

